# Supplementary material for: Picolinic acid is a broad-spectrum inhibitor of enveloped virus entry that restricts SARS-CoV-2 and influenza A virus in vivo
Source: Cell Rep Med. 2023 Jul 17;4(8):101127. doi: 10.1016/j.xcrm.2023.101127 (PMC10439173; doi:10.1016/j.xcrm.2023.101127)
Supplement: Document S1. Figures S1–S6 [file mmc1.pdf]

Cell Reports Medicine, Volume *4*

Supplemental information

Picolinic acid is a broad-spectrum inhibitor of enveloped virus entry that restricts SARS-CoV-2 and influenza A virus *in vivo*

Rohan Narayan, Mansi Sharma, Rajesh Yadav, Abhijith Biji, Oyahida Khatun, Sumandeep Kaur, Aditi Kanojia, Christy Margrat Joy, Raju Rajmani, Pallavi Raj Sharma, Sharumathi Jeyasankar, Priya Rani, Radha Krishan Shandil, Shridhar Narayanan, Durga Chilakalapudi Rao, Vijaya Satchidanandam, Saumitra Das, Rachit Agarwal, and Shashank Tripathi

Supplemental item titles

1. **Fig S1. *In vitro* cytotoxicity of PA and effect of control antivirals against IAV and SARS-CoV-2. Related to Figure 1**
2. **Fig S2. PA inhibits SARS-CoV-2 entry in HEK293T-ACE2 cells. Related to Figure 2**
3. **Fig S3. PA treatment does not inhibit virion binding to the host cell but impedes its endocytic movement toward the nucleus. Related to Figure 3**
4. **Fig S4. PA inhibits ph5.0-induced fusion of IAV with the plasma membrane, does not affect cell membrane architecture but has an irreversible effect on treated virion infectivity. Related to Figure 3**
5. **Fig. S5. *In vivo* toxicity profile and immunomodulatory effects of PA in BALB/c mice. Related to Figure 5**
6. **Fig. S6. *In vivo* toxicity profile of PA in Syrian golden Hamsters and pharmacokinetics of PA in animal lungs and blood plasma. Related to Figure 6**

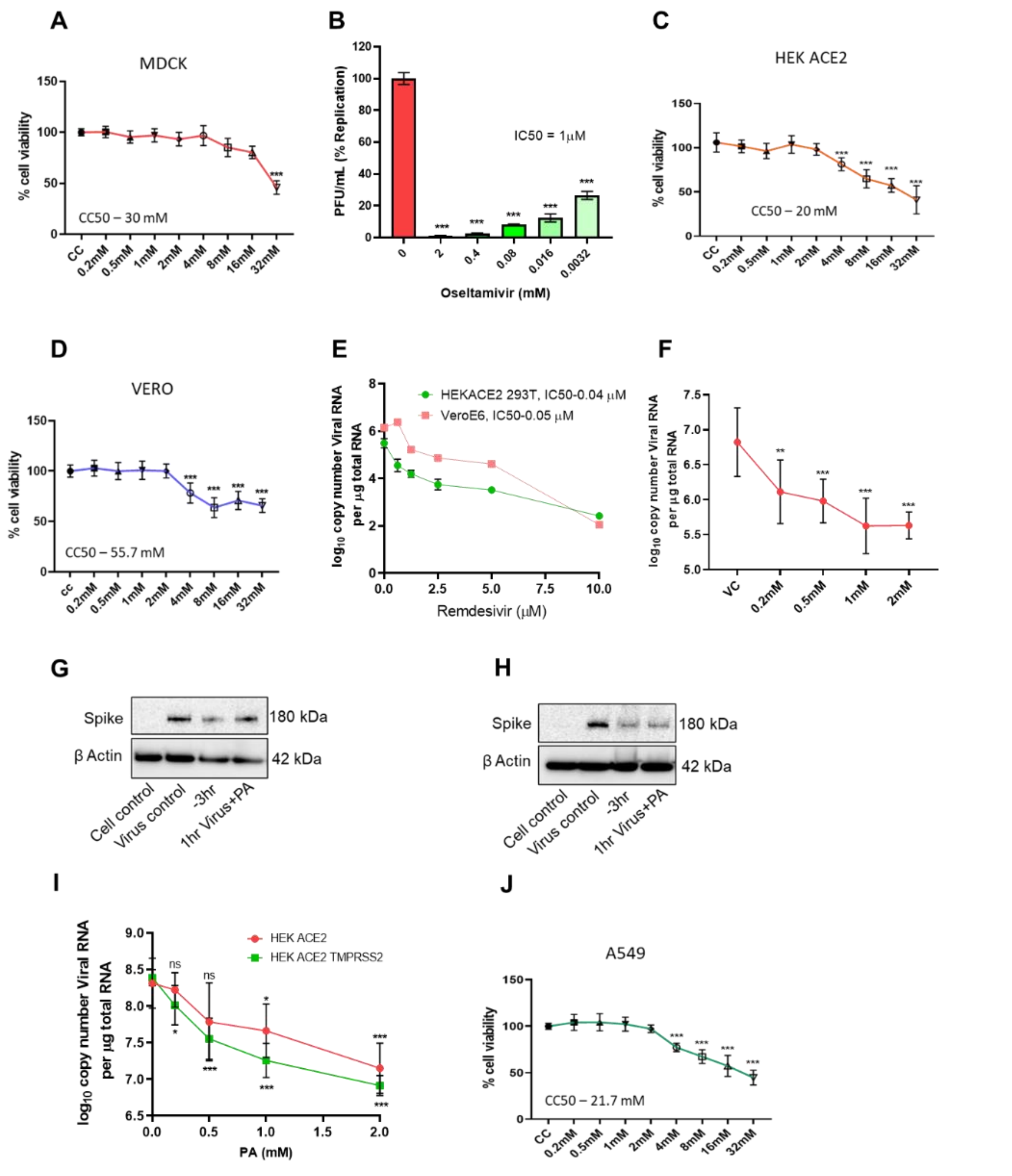

Fig S1. ***In vitro*** **cytotoxicity of PA and effect of control antivirals against IAV and SARS-CoV-2. Related to Figure 1. (A)** MDCK cells were treated with increasing doses of PA as indicated, and cell viability was measured by MTT assay after 48 hr**. (B)** A549 cells were pre-treated for 3 hr with different concentrations of Oseltamivir and infected with 0.001 MOI Cal/09 virus. Infectious virus from cell supernatants was measured by plaque assay. **(C-D)** HEK293T-ACE2 and Vero E6 cells were treated with increasing doses of PA as indicated, and cell viability was measured by MTT assay after 48 hr**. (E)** HEK293T-ACE2 and VeroE6 cells were pre-

treated for 3 hr with increasing concentrations of Remdesivir as indicated and infected with 0.01 MOI (HEK293T-ACE2) or 0.001 MOI (VeroE6). Total cellular RNA was collected at 48 hpi and vRNA was estimated by qRT PCR. **(F)** Caco-2 cells were pre-treated with increasing doses of PA as indicated, infected with 0.01 MOI SARS-CoV-2 and 72 hpi, and vRNA load was estimated by qRT PCR. **(G,H)** HEK293T-ACE2 and HEK293T-ACE2-TMPRSS2 cells were first pre-treated for 3 hr with 2 mM PA (-3hr), infected with 10 MOI SARS-CoV-2 in the presence of the drug, and collected 3 hpi. To test the direct effects of PA on virus particles, the virus inoculum was incubated with 2 mM PA for 1 hr at 37°C and subsequently used for infection (1 hr virus + PA). No additional PA was added here. Viral spike protein expression levels by western blot are shown for (G) HEK293T-ACE2 and (H) HEK293T-ACE2-TMPRSS2 cells. **(I)** HEK293T-ACE2 and HEK293T-ACE2-TMPRSS2 cells were pre-treated with increasing doses of PA as indicated, infected with 0.01 MOI SARS-CoV-2 and 48 hpi, vRNA load was estimated by qRT PCR. **(J)** A549 cells were treated with increasing doses of PA as indicated, and cell viability was measured by MTT assay after 48 hr**.** The data comprises 3 independent biological replicates, with datasets including 2-3 technical replicates. ***$p < 0.001$, using a two-tailed unpaired *t*-test, one-way ANOVA with Dunnett's multiple comparison test, or Brown–Forsythe and Welch ANOVA with Dunnett's T3 multiple comparison test wherever necessary. Error bars represent mean ± SD.

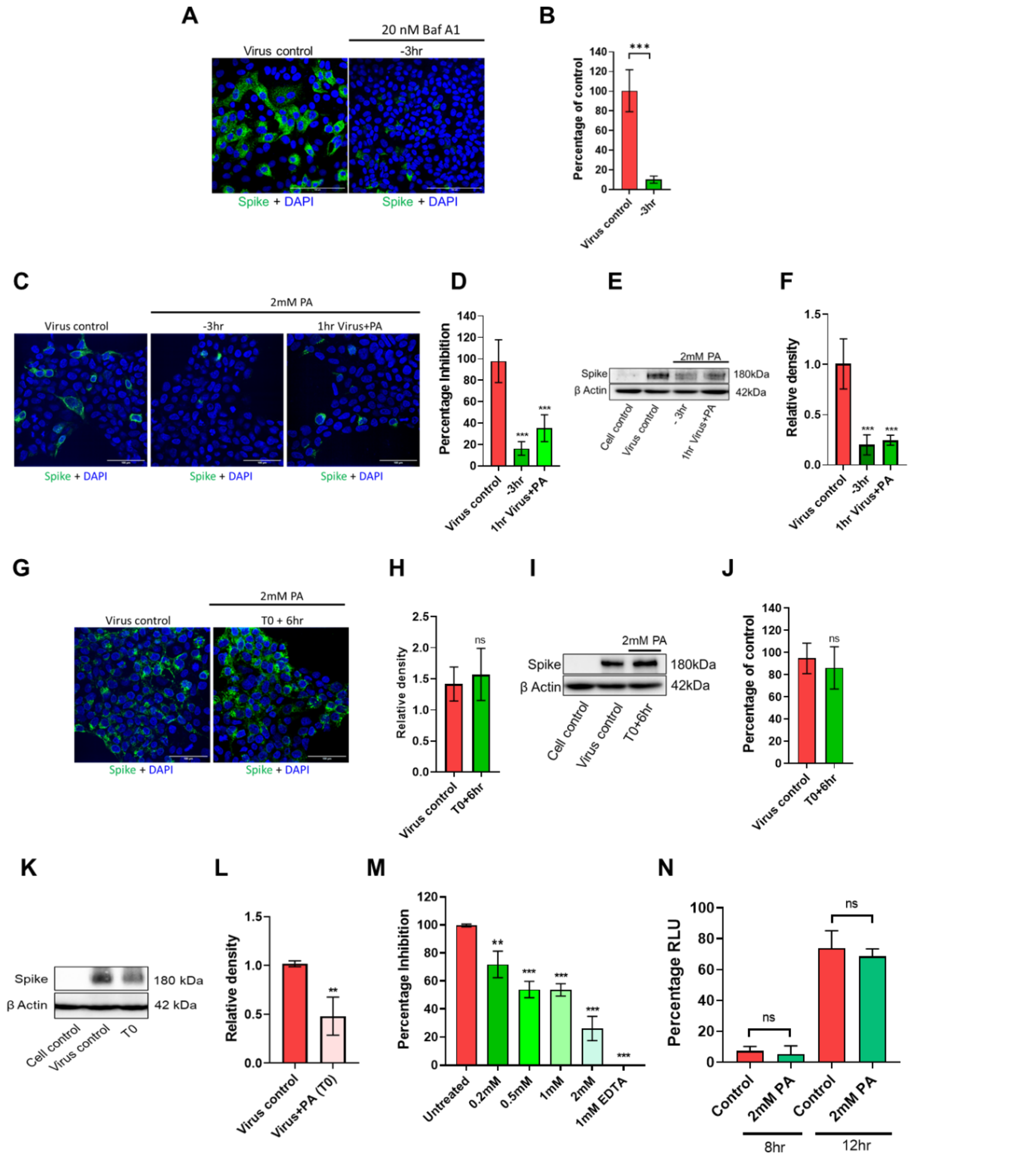

Fig S2. PA inhibits SARS-CoV-2 entry in HEK293T-ACE2 cells. Related to Figure 2. (A-B) Vero E6 cells were pre-treated for 3 hr with 20 nM Bafilomycin A1 and infected with 10 MOI SARS-CoV-2 in the presence of the drug. Cells were then collected at 3 hpi. (A) Confocal images showing viral spike positive cells in green and (B) quantification of cells in green**. (C-F)** Time of addition assay was performed in HEK293T-ACE2 cells by pre-treating cells for 3 hr with 2 mM PA (-3hr), infecting with 10 MOI SARS-CoV-2 in the presence of the drug, and collecting 3 hpi. Alternatively, the virus inoculum was incubated with 2 mM PA for 1 hr at 37°C and subsequently used for infection (1 hr virus + PA). No additional drug was added. (C) Confocal images showing

viral spike positive cells in green and (D) quantification of cells in green. (E) Spike expression levels by western blot for the above conditions and quantification of (F) band intensity. **(G-J)** Untreated HEK293T-ACE2 were first infected with 10 MOI SARS-CoV-2 and treatment with 2 mM PA was done at 6 hpi (T0+6 hr). Cells were then collected 3 hr post addition of the drug. Confocal images with quantification of spike-positive cells and corresponding western blot data are shown in (G,H) and (I,J), respectively. Scale bar = 100µm. (**K, L**) HEK 293T-ACE2 cells were infected with 10 MOI SARS-CoV-2 and treated simultaneously with 2 mM PA. Cell lysates were collected 3 hpi and viral spike protein expression was estimated by (K) western blot analysis and (L) relative band intensity quantified using ImageJ/Fiji. **(M)** HEK293T-ACE2 cells were pre-treated with increasing doses of PA or 1 mM EDTA as indicated, infected with SARS-CoV-2 spike pseudotyped particles, and luciferase expression levels measured 60 hpi **(N)** HEK293T cells were transfected with IAV mini replicon plasmids PA, PB1, PB2, NP, and NP-firefly luc, with pRLTK control. PA (2 mM) was added 3 hr post-transfection, cells were collected 8 and 12 hr later, and luciferase expression was estimated. Data are from 3 independent experiments. **$p < 0.01$, ns – nonsignificant using a two-tailed unpaired *t*-test or one-way ANOVA with Dunnett's multiple comparison test wherever necessary. Error bars represent mean ± SD.

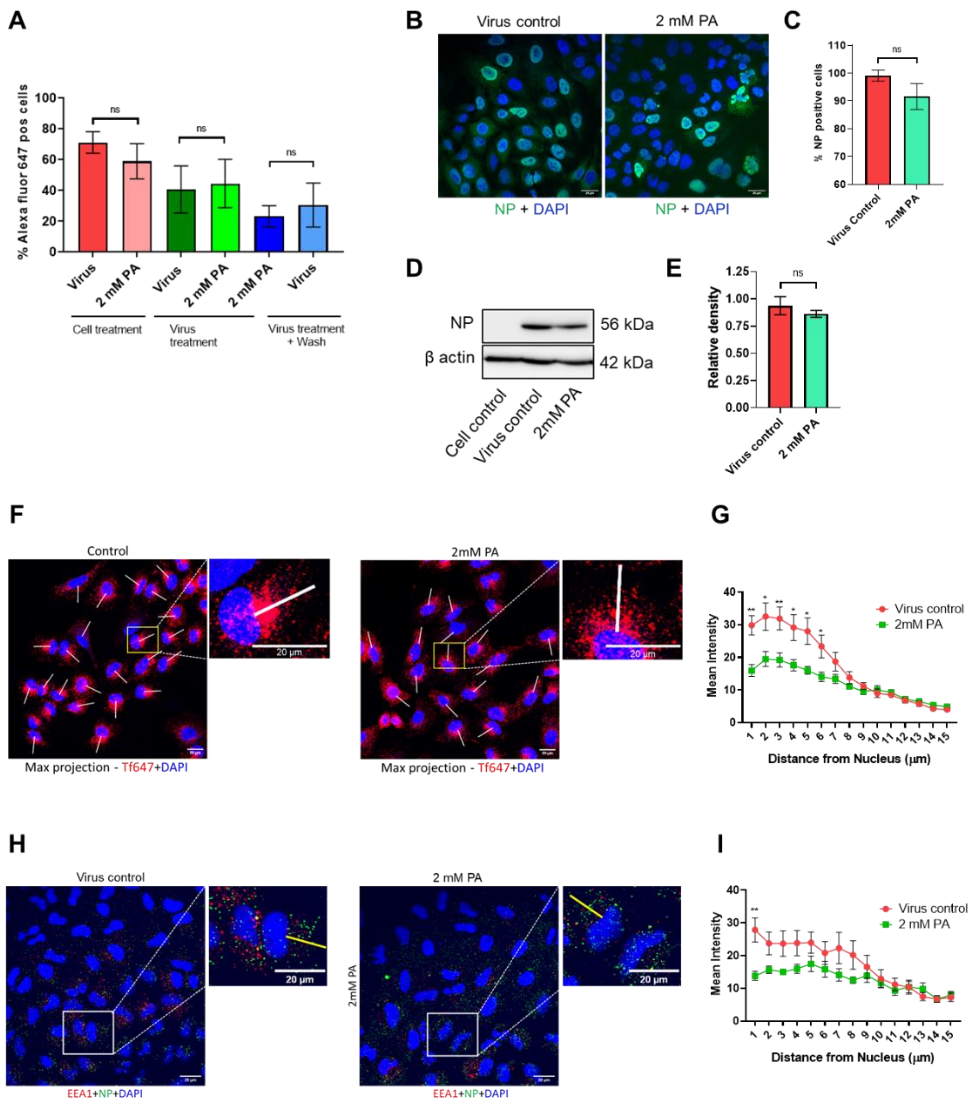

Fig S3. PA treatment does not inhibit virion binding to the host cell but impedes its endocytic movement toward the nucleus. Related to Figure 3. (A) A549 cells suspension or virus inoculum were treated with 2 mM PA under different conditions. Cells were pre-treated for 3hr with 2 mM PA, washed, and infected with 50 MOI PR8 IAV for 60 min on ice in the absence of the drug. Virus inoculum (50 MOI PR8 IAV) was treated with 2 mM PA for 1 hr and used for infection on ice, or the PA-treated inoculum was washed by ultracentrifugation to remove the drug and then used for infection. In all conditions, cells were infected on ice for 1 hr, washed and fixed with 4% PFA and used for HA surface labelling. **(B-E)** A549 cells were infected with 10 MOI PR8 IAV for 1 hr on ice, washed, and warm media containing 2 mM PA was added. After 3 hr, cells were either fixed with 4% PFA for IFA analysis of NP-positive cells, or cell lysates were collected and analyzed by western blot to estimate viral NP expression. (B) Confocal images are showing viral NP-positive cells in green and quantified in (C). (D,E) show corresponding western blot data and quantification of bands, respectively. **(F,G)** A549 cells pre-treated with 2 mM PA were infected with 10 MOI PR8 IAV on ice for 60

min, washed, and incubated at 37°C for another 60 min in the presence of the drug before fixing for IFA. (F) Confocal images show viral NP and cellular EEA-1 in green and red, respectively. (G) Line ROIs were drawn from the nucleus to the cell periphery and fluorescence intensity of Tf-647 was quantified along the length using Leica LasX imaging software. **(H, I)** A549 cells pre-treated for 3 hr with 2 mM PA were pulsed with 25 µg/mL Tf-647 for 30 min, washed, and chased for 15 min in the presence of the drug. (H) Confocal microscopy images showing Tf-647 labeled vesicles in A549 control and 2 mM PA treated cells. (I) Line ROIs were drawn from the nucleus to the cell periphery and the fluorescence intensity of Tf-647 was quantified along the length using Leica LasX imaging software. Scale bar = 20µm. (A) is from 2 independent experiments. All other data are from 3 independent experiments. Datasets include 2-3 technical replicates. *$p < 0.05$, **$p < 0.01$, ns – nonsignificant using a two-tailed unpaired *t*-test or one-way ANOVA with Dunnett's multiple comparison test wherever necessary. Error bars represent mean ± SD.

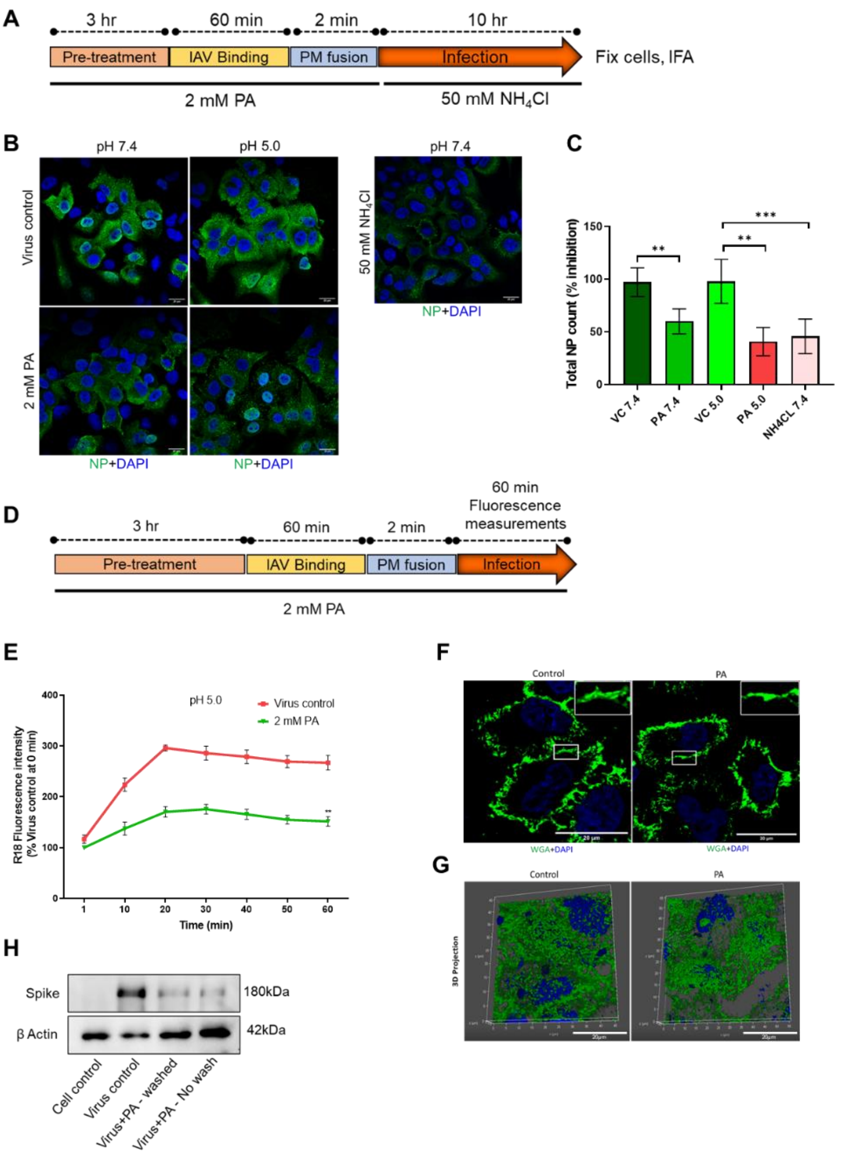

Fig S4. PA inhibits ph5.0-induced fusion of IAV with the plasma membrane, does not affect cell membrane architecture but has an irreversible effect on treated virion infectivity. Related to Figure 3. (A) Schematic showing methodology for endocytosis bypass assay. **(B)** A549 cells pre-treated with 2 mM PA or 50 mM NH_4Cl were infected with 10 MOI PR8 IAV on ice for 60 min. Cells were washed and incubated with either pH 7.4 or pH 5.0 medium for 2 min at 37°C to induce PM fusion. Cells were washed again and incubated for 10 hr with DMEM containing 50 mM NH_4Cl. Negative control of PM fusion used 50 mM NH4Cl at pH 7.4. Cells were then fixed and processed for IFA. Confocal images show NP in green and nuclei in blue. (C) The graph shows the percentage of NP count normalized to virus controls. Quantification was done using ImageJ/Fiji. **(D-E)** R-18 labeled PR8 IAV particles were induced to fuse at the PM in MDCK cells, and the increase in fluorescence intensity upon virus-endosome fusion was measured. (D) Shows a schematic of the

methodology and (E) shows the fluorescence intensity profile up to 60 min. **(F-G)** A549 cells were pre-treated with 2 mM PA for 3hr, fixed with 4% PFA, cell membrane labeled with wheat germ agglutinin (Green), and nuclei with DAPI (Blue). (F) Super-resolution images were acquired using a Leica Stellaris confocal microscope. (G) 3D surface projections created from confocal image z-stacks. Scale bar = 20 μm. **(H)** SARS-CoV-2 virus particles were treated with 2 mM PA for 1 hr at 37°C, washed in PBS for 5 min, ultracentrifuged, and used for infection in Vero E6 cells at 10 MOI. The control virus was not washed and used directly for infection after treatment. Cell lysates were collected at 3 hpi and spike expression was analyzed by western blot. A-E are from 3 independent biological replicates, with datasets including 2-3 technical replicates. F and G are from 1 experiment. H is from 3 independent experiments with technical triplicates. ***$p < 0.001$, **$p < 0.01$ using a two-tailed unpaired *t*-test or one-way ANOVA, wherever necessary. Error bars represent mean ± SD.

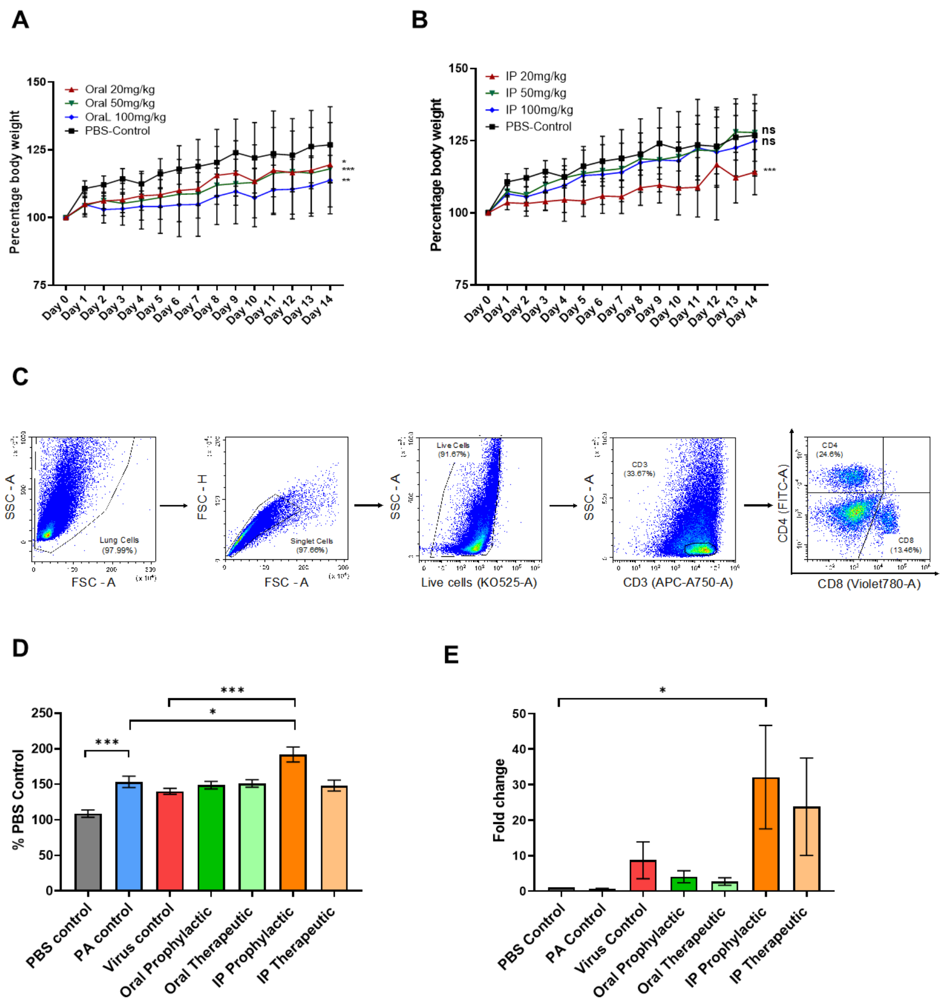

Fig. S5. *In vivo* toxicity profile and immunomodulatory effects of PA in BALB/c mice. Related to Figure 5. Toxicity results as shown by bodyweight changes over 14 days post-treatment with 20, 50, or 100mg/kg PA delivered via **(A)** IP and **(B)** oral routes. Results represent the mean percentage of body weight at Day 0, n= 5 for all groups except n=4 in (A) IP 100mg/kg. **(C-E)** IAV-infected mice, treated with prophylactic and therapeutic dosage regimens of 20 mg/kg PA, were sacrificed 4 dpi, and lungs were analyzed by FACS. (C) Gating strategies to define T cell subsets in lung homogenates. Animal lungs were homogenized, and surface stained with antibodies specific for CD3+, CD4+, and CD8+ markers (D) Quantification of CD4+ T cells from different treatment groups. (E) qRT PCR data showing MIP-1 alpha RNA levels. n=5 in PBS control, PA control, and Oral therapeutic groups and n=4 in all other groups. Data are from 1 experiment. Statistical comparison was done using one-way ANOVA with Bonnferroni's multiple comparisons test. *p< 0.05; **p < 0.01, ***p<0.0001. Error bars indicate mean ± SE.

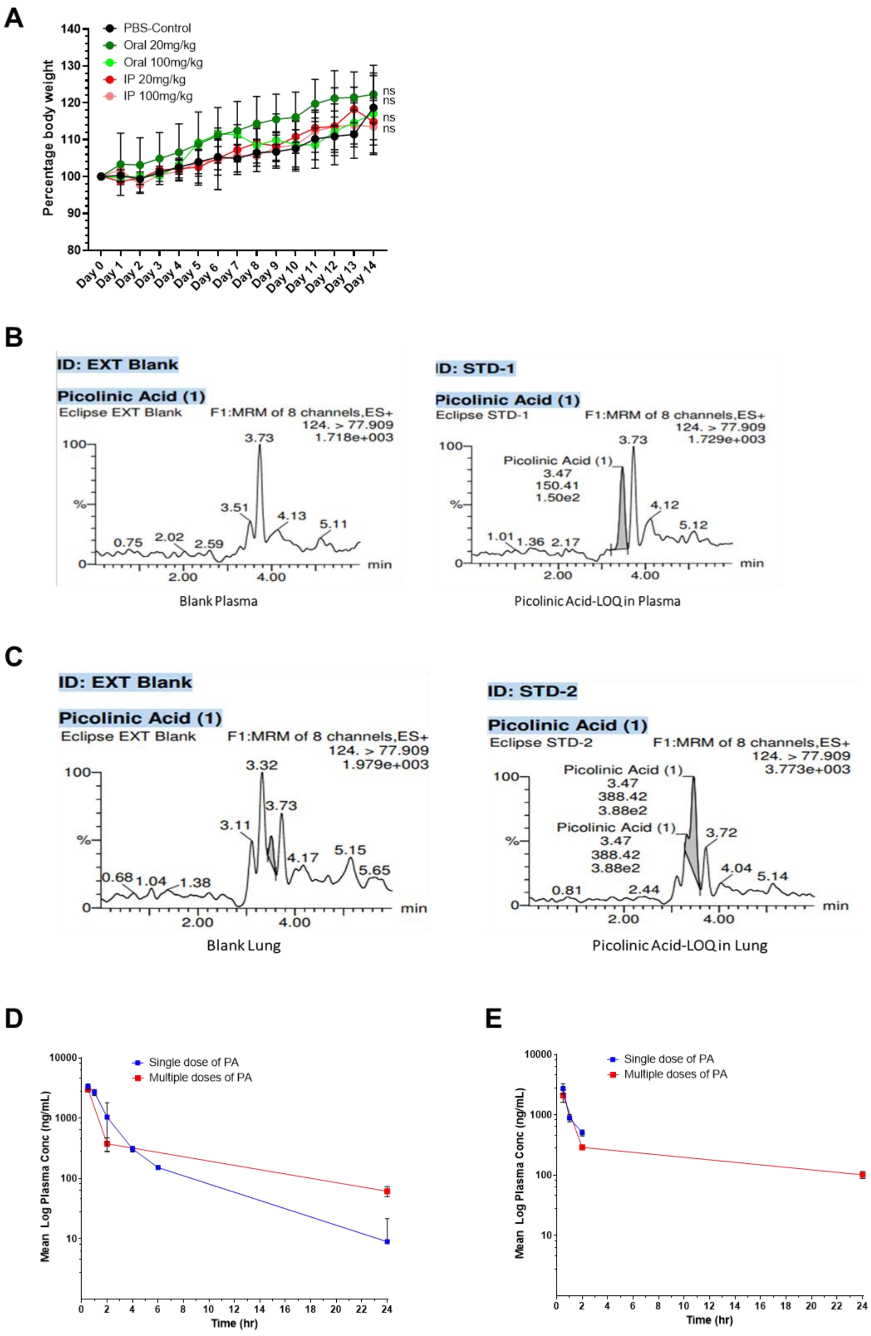

Fig. S6. ***In vivo*** **toxicity profile of PA in Syrian golden Hamsters and pharmacokinetics of PA in animal lungs and blood plasma. Related to Figure 6.** **(A)** 10-12 weeks old mixed gender Syrian golden hamsters were administered with 20 or 100mg/kg BW via IP or oral routes. Bodyweight was monitored up to day 14 post-treatment. Each group includes 2 males and 2 females. **(B-E)** Hamsters were administered with 20 mg/kg PA orally and levels of the drug in plasma and lungs were estimated by Ultra-Performance Liquid Chromatography. Representational blank plasma and lung sample chromatograms showing the PA identity and the quantification limit are shown in **(B)** and **(C)** respectively. Pharmacokinetics of PA in plasma and lungs following single and

multiple oral doses are shown in **(D)** and **(E),** respectively. (B-E) Each group included n=2 animals. The data shown is from 1 experiment. Statistics for A was done using One-way ANOVA with Dunnett's multiple comparisons. ns: nonsignificant. Error bars represent mean ± SD.
