# Supplementary material for: Picolinic acid is a broad-spectrum inhibitor of enveloped virus entry that restricts SARS-CoV-2 and influenza A virus in vivo
Source: Cell Rep Med. 2023 Jul 17;4(8):101127. doi: 10.1016/j.xcrm.2023.101127 (PMC10439173; doi:10.1016/j.xcrm.2023.101127)
Supplement: Document S2. Article plus supplemental information [file mmc4.pdf]

Cell Reports Medicine

Article

Picolinic acid is a broad-spectrum inhibitor of enveloped virus entry that restricts SARS-CoV-2 and influenza A virus *in vivo*

Graphical abstract

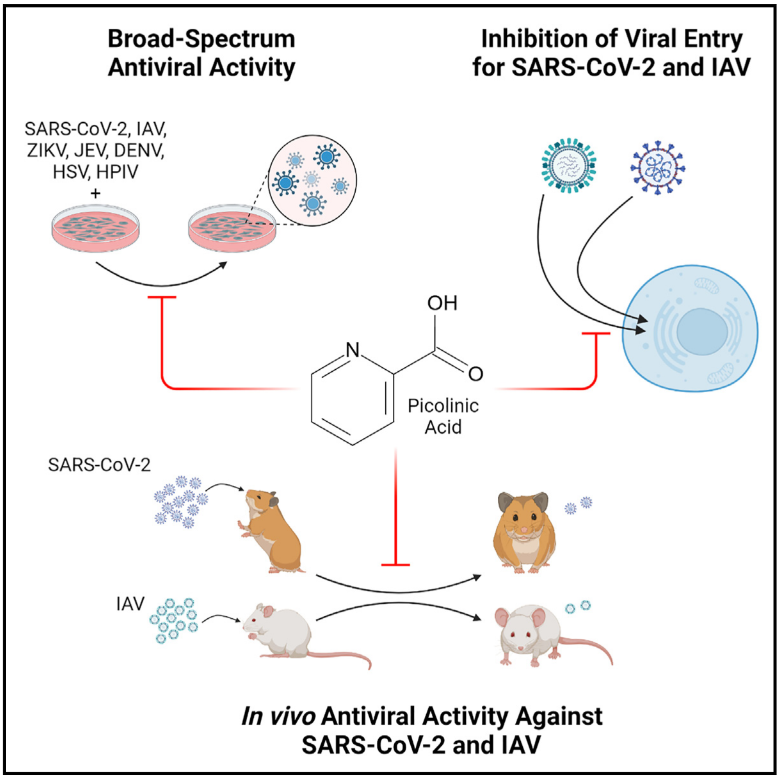

Highlights

- Picolinic acid (PA) is a natural broad-spectrum antiviral against enveloped viruses
- PA inhibits the entry of enveloped viruses by targeting viral-cellular membrane fusion
- PA exhibits antiviral activity against IAV in the murine BALB/c model
- PA exhibits antiviral activity against SARS-CoV-2 in the Syrian golden hamster model

Authors

Rohan Narayan, Mansi Sharma, Rajesh Yadav, ..., Saumitra Das, Rachit Agarwal, Shashank Tripathi

Correspondence

shashankt@iisc.ac.in

In brief

Narayan et al. demonstrate that the naturally occurring tryptophan metabolite picolinic acid is a broad-spectrum antiviral, acting primarily by inhibiting membrane fusion events during viral entry. Also, PA exhibits promising antiviral activity against SARS-CoV-2 and IAV in pre-clinical animal models.

Narayan et al., 2023, Cell Reports Medicine *4*, 101127
August 15, 2023 © 2023 The Author(s).
https://doi.org/10.1016/j.xcrm.2023.101127

CellPress

Cell Reports Medicine

Article

Picolinic acid is a broad-spectrum inhibitor of enveloped virus entry that restricts SARS-CoV-2 and influenza A virus *in vivo*

Rohan Narayan,[1,2] Mansi Sharma,[1,2] Rajesh Yadav,[1,3] Abhijith Biji,[1,2] Oyahida Khatun,[1,2] Sumandeep Kaur,[1,2] Aditi Kanojia,[1,2] Christy Margrat Joy,[1,2] Raju Rajmani,[4] Pallavi Raj Sharma,[3] Sharumathi Jeyasankar,[3] Priya Rani,[2] Radha Krishan Shandil,[6] Shridhar Narayanan,[6] Durga Chilakalapudi Rao,[5] Vijaya Satchidanandam,[2] Saumitra Das,[2] Rachit Agarwal,[3] and Shashank Tripathi[1,2,7,*]

[1]Emerging Viral Pathogens Laboratory, Infosys Wing, Centre for Infectious Disease Research, Indian Institute of Science, Bengaluru 560012, India
[2]Department of Microbiology and Cell Biology, Division of Biological Sciences, Indian Institute of Science, Bengaluru 560012, India
[3]Centre for BioSystems Science and Engineering, Indian Institute of Science, Bengaluru 560012, India
[4]Molecular Biophysics Unit, Indian Institute of Science, Bengaluru 560012, India
[5]Department of Biological Sciences, School of Engineering and Sciences, SRM University, Andhra Pradesh 522240, India
[6]Foundation for Neglected Disease Research, KIADB Industrial Area, Doddaballapur, Bengaluru 561203, India
[7]Lead contact
*Correspondence: shashankt@iisc.ac.in
https://doi.org/10.1016/j.xcrm.2023.101127

SUMMARY

The COVID-19 pandemic highlights an urgent need for effective antivirals. Targeting host processes co-opted by viruses is an attractive antiviral strategy with a high resistance barrier. Picolinic acid (PA) is a tryptophan metabolite endogenously produced in mammals. Here, we report the broad-spectrum antiviral activity of PA against enveloped viruses, including severe acute respiratory syndrome coronavirus 2 (SARS-CoV-2), influenza A virus (IAV), flaviviruses, herpes simplex virus, and parainfluenza virus. Mechanistic studies reveal that PA inhibits enveloped virus entry by compromising viral membrane integrity, inhibiting virus-cellular membrane fusion, and interfering with cellular endocytosis. More importantly, in pre-clinical animal models, PA exhibits promising antiviral efficacy against SARS-CoV-2 and IAV. Overall, our data establish PA as a broad-spectrum antiviral with promising pre-clinical efficacy against pandemic viruses SARS-CoV-2 and IAV.

INTRODUCTION

Emerging and re-emerging viral pathogens pose a serious threat to global public health, and effective broad-spectrum antivirals are critically required to combat them.[1] Direct-acting antivirals targeting viral components are at high risk of viral resistance, especially against RNA viruses.[2] Examples include nirmatrelvir[3] against severe acute respiratory syndrome coronavirus 2 (SARS-CoV-2) main protease and oseltamivir against influenza A virus (IAV) neuraminidase,[4] which can be rendered ineffective quickly due to the emergence of viral resistance. In contrast, host-directed drugs targeting cellular factors essential for the virus life cycle progression pose a higher resistance barrier.[5] Cellular entry is a critical host-dependent step of the viral life cycle. There are limited modes of viral entry, which are often shared among different viruses. Several major human viral pathogens, especially those with pandemic potential, are enveloped, e.g., coronaviruses, influenza viruses, retroviruses, flaviviruses, and herpes viruses.[6] Fusion of viral-cellular membranes is a shared feature of enveloped virus entry and hence an attractive target for broad-spectrum antiviral development.[7] Picolinic acid (PA), also known as pyridine-2-carboxylic acid or 2-picolinic acid (PubChem, CID 1018) (Figure 1A), is a naturally occurring metabolite produced during the catabolism of tryptophan via the kynurenine pathway.[8,9] PA was recently shown to impact endosome maturation, a common viral entry pathway[10] and hence a target for broad-spectrum antiviral development.[11] Considering this, we examined the effect of PA on a range of human viral pathogens of clinical significance, including SARS-CoV-2 and IAV, dengue virus (DENV), Zika virus (ZIKV), Japanese encephalitis virus (JEV), herpes simplex virus (HSV), human parainfluenza virus (HPIV), rotavirus, and coxsackie virus (CSV). Mechanism of action (MoA) studies revealed that PA selectively inhibits the entry of enveloped viruses. It does so by targeting viral membrane integrity, preventing viral-cellular membrane fusion, and impeding endocytic vesicle trafficking. Unsurprisingly, PA was ineffective against non-enveloped viruses and bacteriophages. More importantly, PA showed promising pre-clinical antiviral efficacy against SARS-CoV-2 in the Syrian hamster model and against IAV in BALB/c murine model. Overall, this study paves the way for further clinical development and use of PA as a broad-spectrum antiviral, which can help in combating

This is an open access article under the CC BY license (http://creativecommons.org/licenses/by/4.0/).

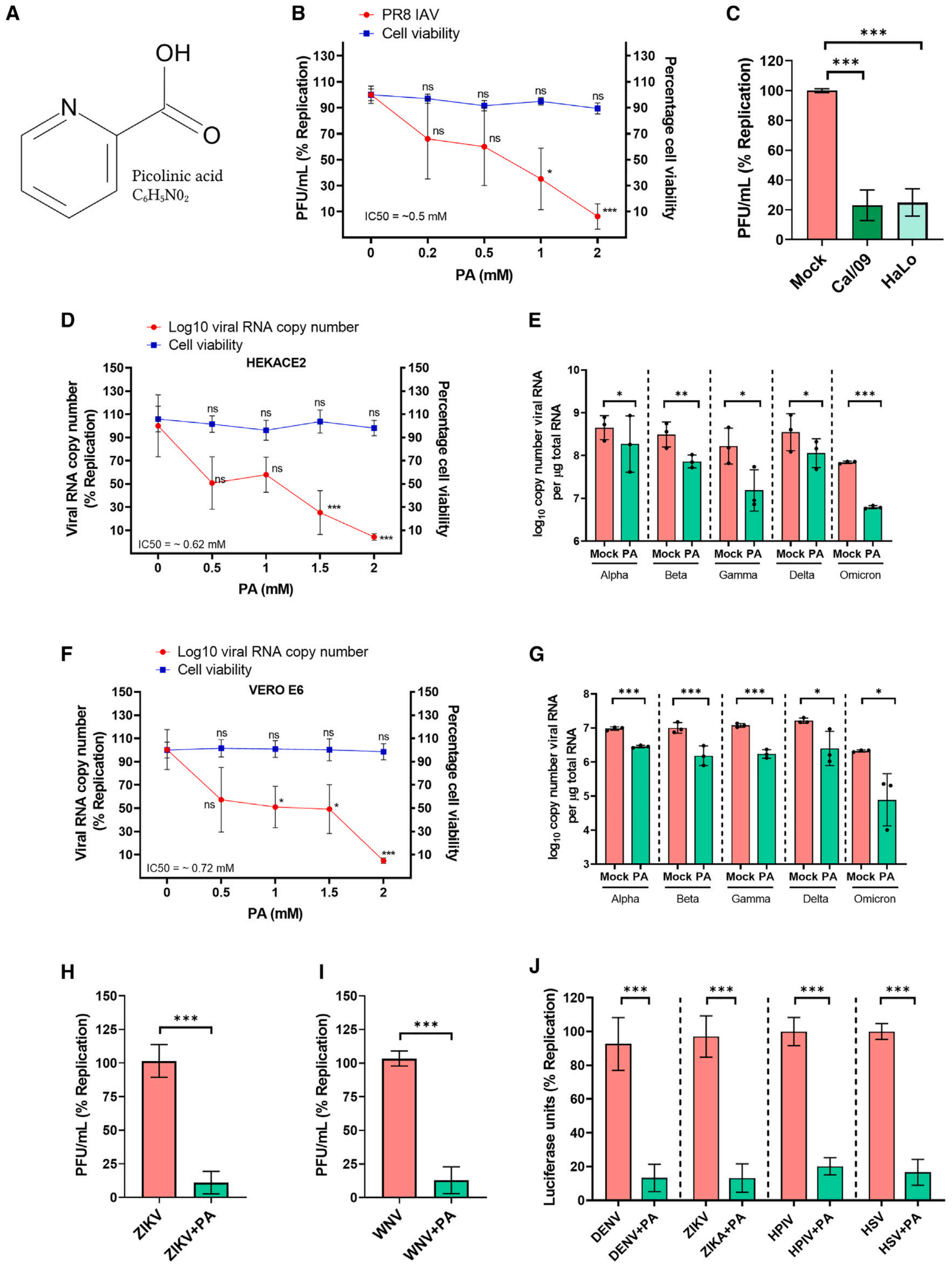

Figure 1. PA exhibits broad-spectrum antiviral activity against enveloped viruses

(A) The chemical structure of picolinic acid (PA).

(B) MDCK cells were pre-treated with increasing PA concentrations, infected with 0.001 MOI PR8 IAV. Results show percentage of infectious virus from supernatants 48 hpi quantified by plaque assay, along with cytotoxicity of the drug concentrations used.

(C) Plaque assay results for 2 mM PA-treated MDCK cells infected with 0.001 MOI Cal/09 or HaLo IAV.

(legend continued on next page)

the COVID-19 pandemic and other emerging and re-emerging enveloped viruses.

RESULTS

PA exhibits broad-spectrum antiviral activity against human-enveloped viruses

In a recent study, PA was shown to interact with the cellular E3 ligase UBR4 and interfere with endosome maturation.[10] Since endocytic processes are co-opted for cellular entry by various viruses, we reasoned that PA must exhibit broad-spectrum antiviral activity. To this end, we first tested PA on H1N1 IAV PR8 (A/Puerto Rico/8/1934 H1N1) replication in Madin-Darby canine kidney (MDCK) cells and observed a dose-dependent decrease in infectious virus counts, with ~95% inhibition at 2 mM PA (IC50: 0.5 mM), which was non-toxic to cells (cytotoxic concentration [CC50] = 30 mM, selectivity index [SI]: (CC50/IC50) = 60) (Figures 1B and S1A). For comparison, oseltamivir inhibited PR8 IAV at an IC50 of 1 μM (Figure S1B). Next, we examined the antiviral effect of PA against the 2009 H1N1 "swine flu" pandemic IAV (Cal/09) and H5N1 IAV (HALo) and observed a consistent inhibitory effect in MDCK cells (Figure 1C). Further on, we tested PA against SARS-CoV-2 isolate Hong Kong/VM20001061/2020 (hereby referred to as SARS-CoV-2) in human HEK293T-ACE2 cells and observed a ~99% reduction in viral RNA (vRNA) load at a non-toxic dose of 2 mM PA (Figures 1D and S1C). When tested against four SARS-CoV-2 variants of concern (VOCs) in this cell line, PA reduced vRNA levels by ~1 log10 units for Gamma and Omicron variants and <1 log10 for other VOCs (Figure 1E). These findings were also validated in non-human primate origin Vero E6 cells (SI = 77) (Figures 1F, 1G, and S1D). For comparison, the SARS-CoV-2 RNA-dependent RNA polymerase (RdRp) inhibitor remdesivir caused a ~4 log10 reduction in SARS-CoV-2 vRNA load at IC50–0.05 μM in both these cell lines (Figure S1E).

Next, we tested the antiviral effects of PA in a cell line expressing TMPRSS2, wherein virus entry can occur via cell membrane fusion. For this, we performed multicycle infection in Caco-2 cells, which constitutively express TMPRSS2,[12] and results showed ~1 log10 reduction of vRNA load 72 h post-infection (hpi) in the presence of 1 and 2 mM PA (Figure S1F). To further elucidate PA's effect on viral fusion at the cell membrane, we made the HEK293T-ACE2-TMPRSS2 cell line and performed experiments in these cells in parallel with HEK293T-ACE2 cells. The 3 h time of addition (ToA) experiments indicated inhibition of virus entry in both cell lines (Figures S1G and S1H). However, in a multicycle infection, we observed that PA had a more substantial inhibitory effect on HEK293T-ACE2-TMPRSS2 cells (Figure S1I).

Further, we investigated the potential broad-spectrum effects of PA by testing it against the flaviviruses ZIKV and West Nile virus (WNV) in the human lung epithelial cell line A549 and observed ~90% reduction in infectious virus load at a non-toxic dose (Figures 1H, 1I, and S1J). Similar levels of inhibition were observed upon testing against DENV, ZIKV, HPIV, and HSV reporter viruses expressing luciferase in A549 cells, where PA treatment resulted in 80%–90% viral inhibition (Figure 1J). With this set of experiments, the broad-spectrum antiviral activity of PA against a range of human-enveloped viruses was confirmed *in vitro.*

PA inhibits the entry of enveloped viruses into the host cell

To understand the MoA of PA, we performed ToA assays.[13] For IAV, 2 mM PA pre-treatment of A549 cells for 3 h or the virus for 1 h reduced infection significantly (Figures 2A–2D). Introducing PA during post-entry stages of virus replication (6 hpi) did not affect infection (Figures 2E–2H). ToA experiments in PA-treated Vero E6 cells infected with SARS-CoV-2 also showed data consistent with the inhibition of virus entry (Figures 2I–2L). As a control, we used the V-ATPase inhibitor bafilomycin A1, which is known to affect SARS-CoV-2 entry in Vero cells,[14] and observed a >90% reduction in spike signal (Figures S2A and S2B). We also performed ToA experiments in HEK293T-ACE2 cells and observed similar effects of PA on SARS-CoV-2 entry as in Vero E6 cells (Figures S2C–S2F). In both cell lines, the introduction of PA post-virus entry at 6 hpi did not affect infection (Figures 2M–2P and S2G–S2J). Moreover, in the absence of pre-treatment, when cells were only treated at the time of infection, there was a partial reduction in viral spike expression (Figures S2K and S2L). We also validated the effect of PA on virus entry using SARS-CoV-2 spike pseudotyped particles expressing luciferase, which is indicative of virus entry inhibition.[15] Here, we observed a dose-dependent effect of PA, with 90% inhibition of luciferase expression at 2 mM concentration in HEK292T ACE2 cells. For comparison, we also used 1 mM ethylenediaminetetraacetic acid (EDTA) treatment of cells to test the effects of a metal chelator on spike pseudotyped virus entry. Results showed almost complete inhibition of virus entry in the presence of EDTA (Figure S2M). Furthermore, we used a mini replicon assay to examine the direct effects of PA on viral (IAV) polymerase activity, and results indicated that 2 mM PA did not affect the same (Figure S2N).

PA inhibits enveloped viral entry by blocking viral-cellular membrane fusion

To delineate which specific step of viral entry is targeted by PA, we first explored the effects of PA on virus binding to the cell

(D) HEK293T-ACE2 cells were pre-treated for 3 h with increasing doses of PA as indicated and infected with 0.01 MOI SARS-CoV-2. Cells were collected at 48 hpi, and vRNA copy was estimated by qRT-PCR and plotted as a percentage of viral replication, with cytotoxicity.

(E) Similar data in HEK293T-ACE2 cells using five SARS-CoV-2 VOCs treated with 2 mM PA, plotted as log10 vRNA copy number.

(F and G) Datasets corresponding to Vero E6 cells infected with 0.001 MOI.

(H and I) A549 cells were pre-treated with 2 mM PA for 3 h and infected with 0.1 MOI ZIKV or WNV. After 48 h, the infectious virus from the supernatant was quantified by plaque assay, and results are shown in (H) and (I).

(J) A549 cells pre-treated with 2 mM PA were infected with different luciferase reporter viruses as indicated. Cells were harvested at 48 hpi, and luciferase expression was quantified using a TECAN plate reader.

The data comprise 3 independent biological replicates, with datasets including 2–3 technical replicates. *$p < 0.05$; **$p < 0.01$; ***$p < 0.001$; ns, non-significant, using Brown-Forsythe and Welch ANOVA with Dunnett's T3 multiple comparison test or unpaired t test with Welch's correction, wherever necessary. Error bars represent mean ± standard deviation (SD).

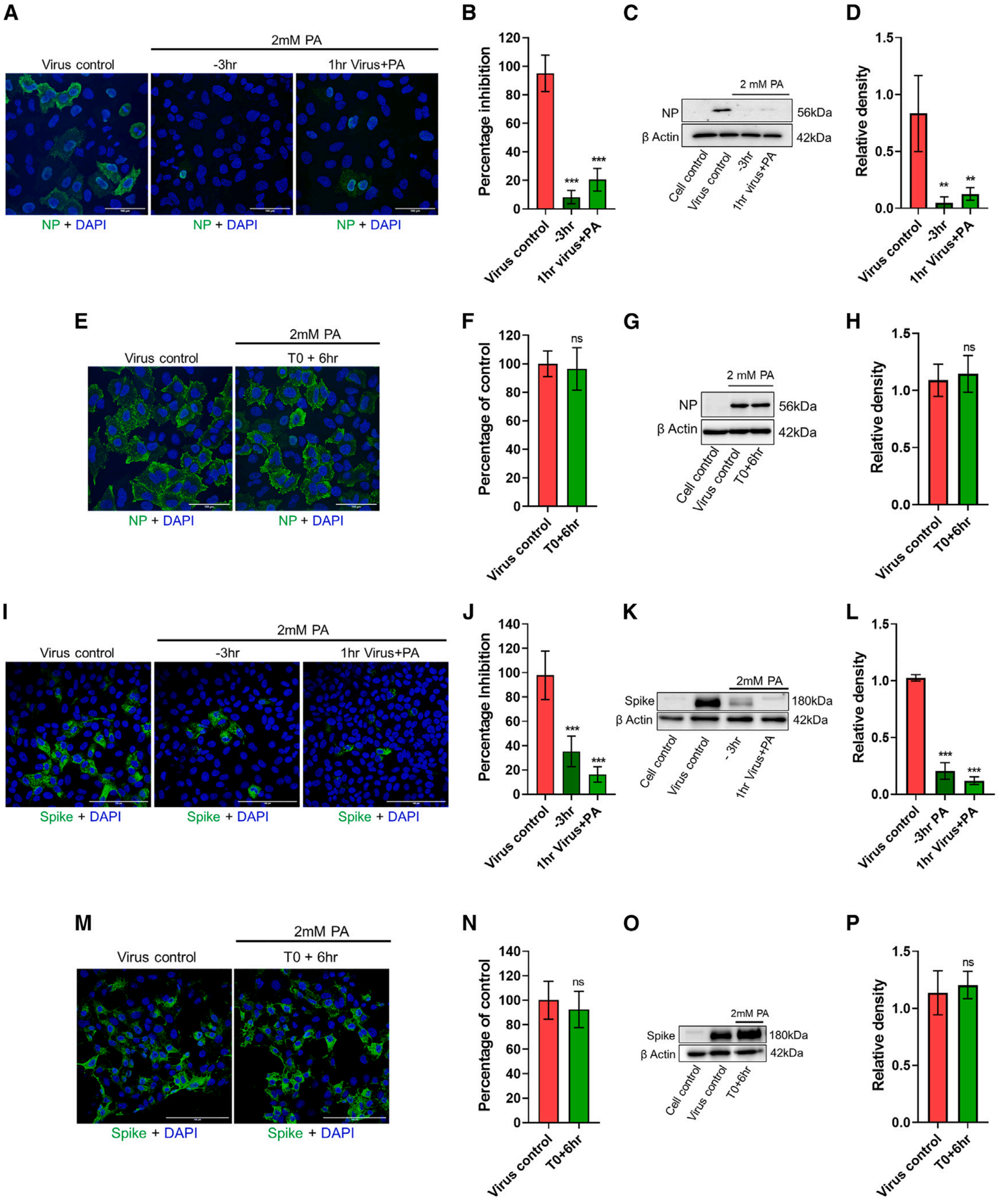

Figure 2. PA inhibits entry of influenza A virus and SARS-CoV-2 into host cells

(A–D) In the time of addition assay, A549 cells were first pre-treated for 3 h with 2 mM PA (−3 h), infected with 5 MOI PR8 influenza A (IAV) in the presence of the drug, and collected 3 hpi. To test the direct effects of PA on virus particles, the virus inoculum was incubated with 2 mM PA for 1 h at 37°C and subsequently used for infection (1 h virus+PA). No additional PA was added here.

(A and B) Confocal images showing viral NP-positive cells in green (A), and quantification of green cells (B).

(C and D) Viral NP expression levels by western blot (C) and quantification of bands (D).

(E–H) Untreated A549 cells were first infected with 5 MOI PR8 IAV, and treatment with 2 mM PA was done at 6 hpi (T0+6 h). Cells were then collected 3 h post-addition of the drug. Confocal images with quantification and western blot data are shown in (E) and (F) and (G) and (H).

(I–P) Entry assays using 10 MOI SARS-CoV-2 in Vero E6 cells were followed as for IAV. Scale bar: 100 μm.

The data comprise 3 independent biological replicates, with datasets including 2–3 technical replicates. $^{**}p < 0.01$, $^{***}p < 0.001$; ns, non-significant using two-tailed unpaired t test or Brown-Forsythe and Welch ANOVA with Dunnett's T3 multiple comparison tests, where applicable. Error bars represent mean ± SD.

CellPress
OPEN ACCESS

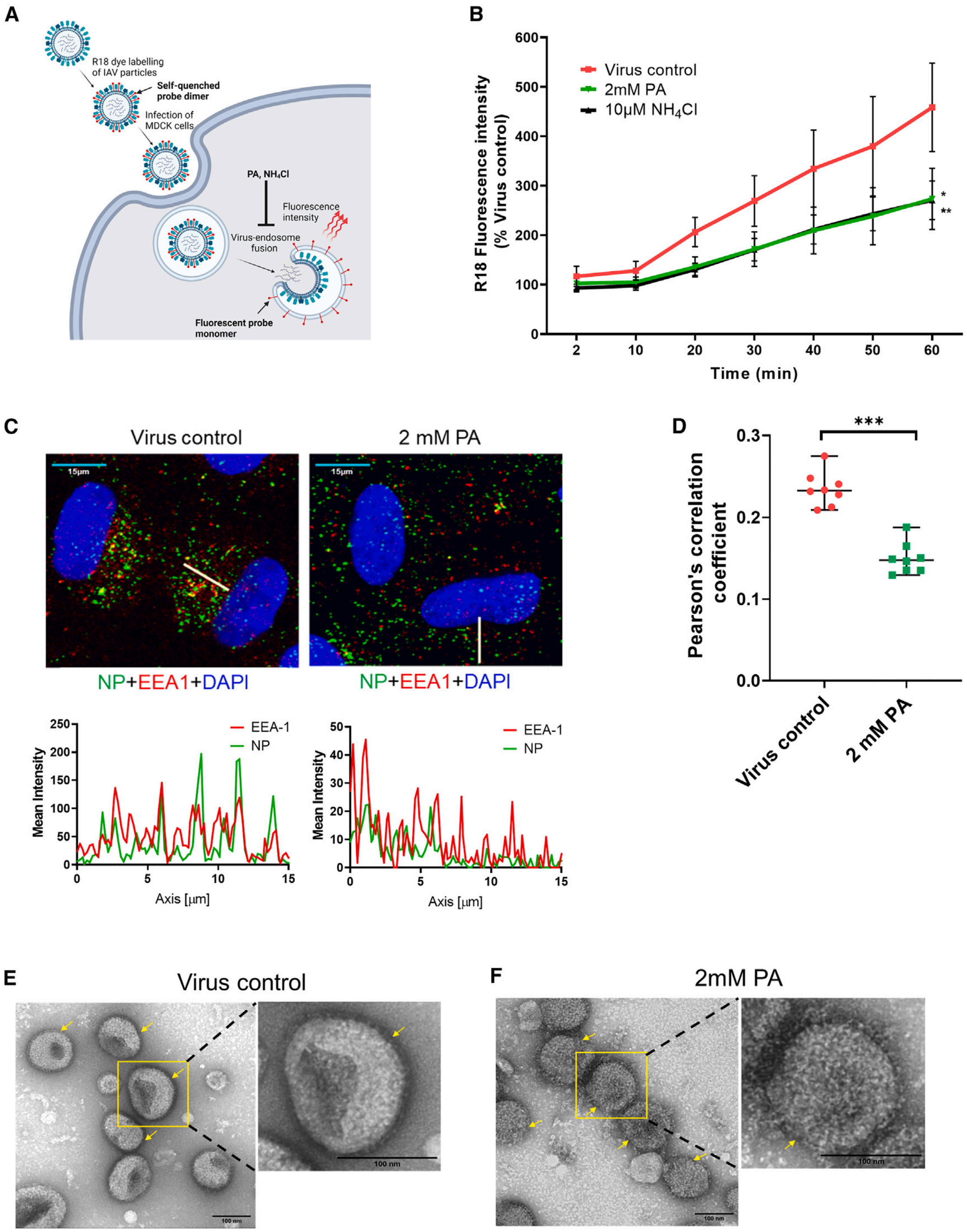

Figure 3. PA acts by targeting viral membrane and inhibiting viral-cellular membrane fusion

(A) Schematic of virus-endosome fusion assay based on fluorescence self-quenching. Fluorescence associated with R18 probe-labeled virus particles is quenched due to dye-dye interactions. Upon virus entry, virus membrane fusion mediated by a drop in endosomal pH causes dispersion of the probe, resulting in increased fluorescence intensity due to de-quenching mediated by monomerization of the probe.

(B) R18-labeled PR8 IAV particles were used to infect MDCK cells, and the increase in fluorescence intensity upon virus-endosome fusion was measured.

(C and D) A549 cells pre-treated with 2 mM PA were infected with 10 MOI PR8 IAV on ice for 60 min, washed, and incubated at 37°C for another 60 min in the presence of the drug before fixing for IFA.

(C) Confocal images show viral NP and cellular EEA-1 in green and red, respectively. The fluorescence intensity profile along the line regions of interest (ROIs; white) drawn from the nuclei was generated using Leica LAS X software (scale bar, 15 μm).

(legend continued on next page)

surface by its direct effects on cells or virus particles. Pre-treatment of cells for 3 h did not have any consequence on virus binding to cells, as shown by influenza virus hemagglutinin (HA) surface labeling (Figure S3A). Also, no effects on viral entry were observed upon treatment of virus particles with PA, with or without washing off the drug from the virus preparation (Figure S3A). When PA was introduced after allowing the virus to bind to the cell surface on ice, no significant reduction in infection was observed, as shown by immunofluorescence assay (IFA) images and western blot analysis (Figures S3B–S3E). Overall, the data did not show any effects of PA on virus binding.

Further on, we used IAV and SARS-CoV-2 models to explore the effects of PA on membrane fusion. During IAV entry, viral-cellular membrane fusion occurs inside the endosomal compartment due to pH-dependent conformational changes triggered in the HA protein.[16] To test the effect of PA on this, we used octadecyl rhodamine B (R18) fluorescent probe-labeled IAV virions.[17] In intact virions, the dye signal is quenched; however, the dye is de-quenched during viral entry, and fluorescence intensity increases, indicating the fusion of viral and endocytic membranes (Figure 3A). We observed that PA inhibited viral-endocytic membrane fusion in pre-treated MDCK cells comparable to the well-known pH-dependent endocytic viral entry inhibitor ammonium chloride (NH_4Cl)[18] (Figure 3B). In an earlier study, PA has been reported to interfere with endocytic vesicle maturation.[10] To confirm, we treated A549 cells with PA and incubated them with Transferrin Alexa Fluor 647 conjugate (Tf 647). We observed that in control cells, Tf 647-loaded vesicles had predominantly perinuclear localization, which, in the case of PA-treated cells, was dispersed across the cytoplasm (Figures S3E and S3F). A similar effect was observed on the distribution of incoming IAV virions in A549 cells. Influenza virus entry occurs via clathrin-mediated endocytosis, and the virus particles traffic through early and late endosomes before initiating pH-dependent fusion with the endosomal membrane.[19,20] EEA-1 (early endosome antigen-1)-labeled endosomes are widely used for influenza virus entry studies,[21,22] and considering the known effects of PA on endosome biogenesis,[10] we used this marker to understand the early events that transpire upon IAV entry. We observed that at 60 min post-infection, much of the internalized nucleoprotein (NP)-labeled IAV particles were in the perinuclear region, compared with a more scattered phenotype of the endocytic vesicles in the presence of PA (Figures 3C and S3G–S3H). Analysis of Pearson's correlation coefficient showed significantly reduced co-localization between NP signal from incoming IAV particles and EEA-1-positive early endosomes upon PA treatment (Figure 3D).

Furthermore, we tested the effect of PA on viral fusion at the plasma membrane (PM). For this, we used a method[23] where the entry inhibitor drug remains in the medium up to the fusion step, after which cells are incubated for 10 h in the presence of NH_4Cl, which prevents entry of unfused viruses that may have entered via endocytosis (Figure S4A). Results showed that the total IAV NP count in PA-treated cells was significantly reduced both at pH 7.4 and 5, indicating that PA abrogates IAV entry via both inhibition of virus endocytosis and membrane fusion (Figures S4B and S4C). The effect of PA at pH 7.4 was comparable to that of NH_4Cl control (Figures S4B and S4C). To further corroborate these results, we used the pH 5 condition to induce R18-labeled IAV-PM fusion and monitored the increase in fluorescence over time. Results showed decreased fluorescence upon PA treatment, indicating inhibition of virus fusion with the PM as well (Figures S4D and S4E). Finally, to elucidate the mechanistic basis of viral-cellular membrane fusion inhibition by PA, we examined PA-treated IAV virions by transmission electron microscopy (TEM). The images showed disruption of the IAV membrane upon PA treatment, although viral glycoproteins were still intact (Figures 3E and 3F). On the other hand, PA treatment did not seem to affect the cell membrane integrity, as observed by fluorescently labeled wheat germ agglutinin (WGA-488) staining of PA-treated cells (Figures S4F and S4G; Videos S1 and S2). However, the effect of PA on the viral envelope was irreversible, as washing the PA off through ultracentrifugation could not restore the SARS-CoV-2 virion infectivity (Figure S4H). Taken together, these data suggest that the primary action of PA against IAV and SARS-CoV-2 is a combination of viral envelope disruption with inhibition of viral entry or early events, including viral-cellular membrane fusion.

PA is ineffective against non-enveloped viruses, including bacteriophages

So far, we have confirmed the broad-spectrum antiviral activity of PA against enveloped viruses, which involved targeting viral envelope. We reasoned that viruses that do not have a host-derived lipid envelope should be less sensitive to PA. To test this, we examined PA's effect on infection by a panel of non-enveloped viruses. To begin, we tested PA's effect on coxsackievirus B3 (CVB3) infection in HeLa cells and observed that pre-treatment of cells, incubation of the virus with the PA before infection, and treatment at the time of infection did not have any effects on the virus entry or multicycle replication (Figures 4A–4C). Thereafter, we examined the effect of PA on Rhesus monkey rotavirus (RRV) infection in HEK293T cells, and here also it did not inhibit virus infection, measured by quantification of viral protein 6 (VP6)-positive cells (Figures 4D and 4E). Next, we used adenovirus 5 particles expressing EGFP (Ad5-EGFP) to infect HEK293T cells, and here, PA treatment showed no effect on GFP-positive cell count (Figure 4F). In a similar experiment, we evaluated PA's effects on an adeno-associated virus expressing EGFP (AAV6-153 EGFP), and in this case, 2 mM PA resulted in a statistically insignificant decrease in GFP-positive cell count (Figure 4G). All the viruses tested so far infect humans or higher vertebrates. We then examined the antiviral activity of PA against bacteriophage. For this, we used TM4 mycobacteriophage and *M. smegmatis* as

(D) Shows Pearson's correlation coefficients calculated from red and green channels.

(E and F) TEM images for PR8 IAV particles treated with 2 mM PA for 1 h. Arrows indicate differences in viral membrane integrity between control and PA treatment.

Statistics for (B) used one-way ANOVA with Dunnett's multiple comparisons test at 60 min time point. An unpaired t test comparing medians was used in (D). The data comprise 3 independent biological replicates, with datasets including 2–3 technical replicates. Error bars represent mean ± SD.

CellPress
OPEN ACCESS

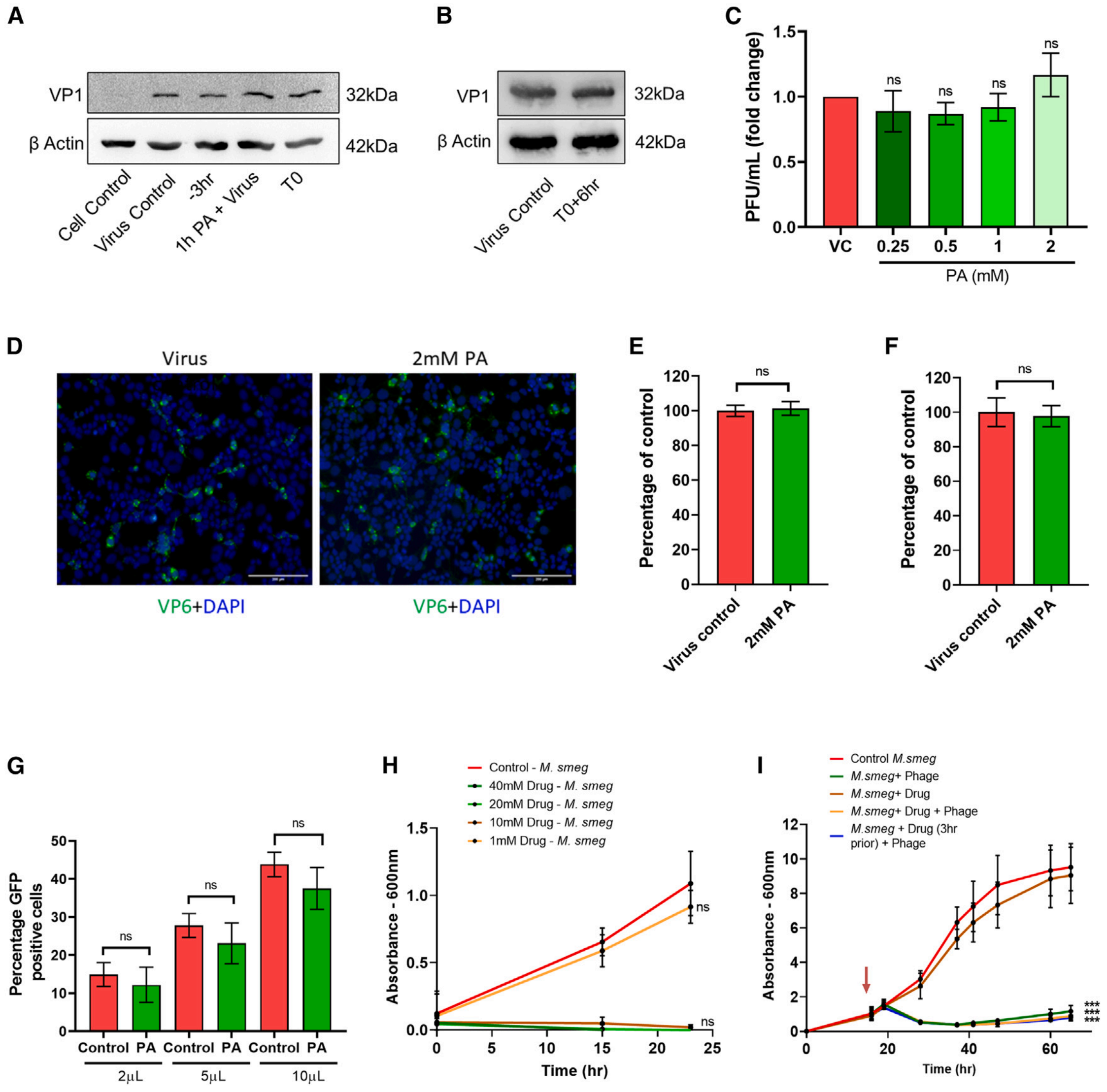

Figure 4. PA does not affect infection by non-enveloped viruses

(A) HeLa cells were either pre-treated for 3 h with 2 mM PA, infected with 10 MOI CVB3, and collected 3 h later (−3 h); treated during infection (T0); or virus and drug were incubated for 1 h and used for infection (1 h virus+PA). Data show CVB3 VP1 expression by western blot.

(B) HeLa cells were infected with 10 MOI CVB3, PA treatment was done 6 hpi (T0+6 h), and cells were collected after 3 h. CVB3 VP1 expression by western blot is shown.

(C) HeLa cells were pre-treated with increasing concentrations of PA and infected with 0.1 MOI CVB3, and 48 h later, the virus in supernatants was quantified by plaque assay.

(D and E) HEK293T cells pre-treated with 2 mM PA were infected with RRV and, 12 hpi, were fixed and immunolabeled with rotavirus VP6 antibody. (D) shows representative IFA images showing virus-infected cells in green (scale bar, 200 μm), and percentage of infected cells was quantified by flow cytometry in (E).

(F) HEK293T cells pre-treated with 2 mM PA were infected with 10 MOI Ad5-CMV-hACE2/RSV EGFP and harvested 24 h later, and GFP-positive cells were quantified by flow cytometry.

(G) HEK293T cells pre-treated with 2 mM PA were infected with AAV6-EGFP particles in the presence of the drug at different volumes as indicated. After 48 h, GFP-positive cells were quantified by flow cytometry.

(H) *M. smegmatis* cells in a 48-well plate were treated with increasing concentrations of PA as indicated, and OD600 measurements were taken up to 24 h.

(I) Log-phase secondary bacterial cultures were treated with 1 mM PA at regular time intervals as indicated and infected with 10 MOI TM4 mycobacteriophage. OD600 measurements were taken up to 60 h.

The data comprise 3 independent biological replicates, with datasets including 2–3 technical replicates. ***$p < 0.001$; ns, non-significant using two-tailed unpaired t test or one-way ANOVA with Dunnett's multiple comparisons wherever applicable. Error bars represent mean ± SD.

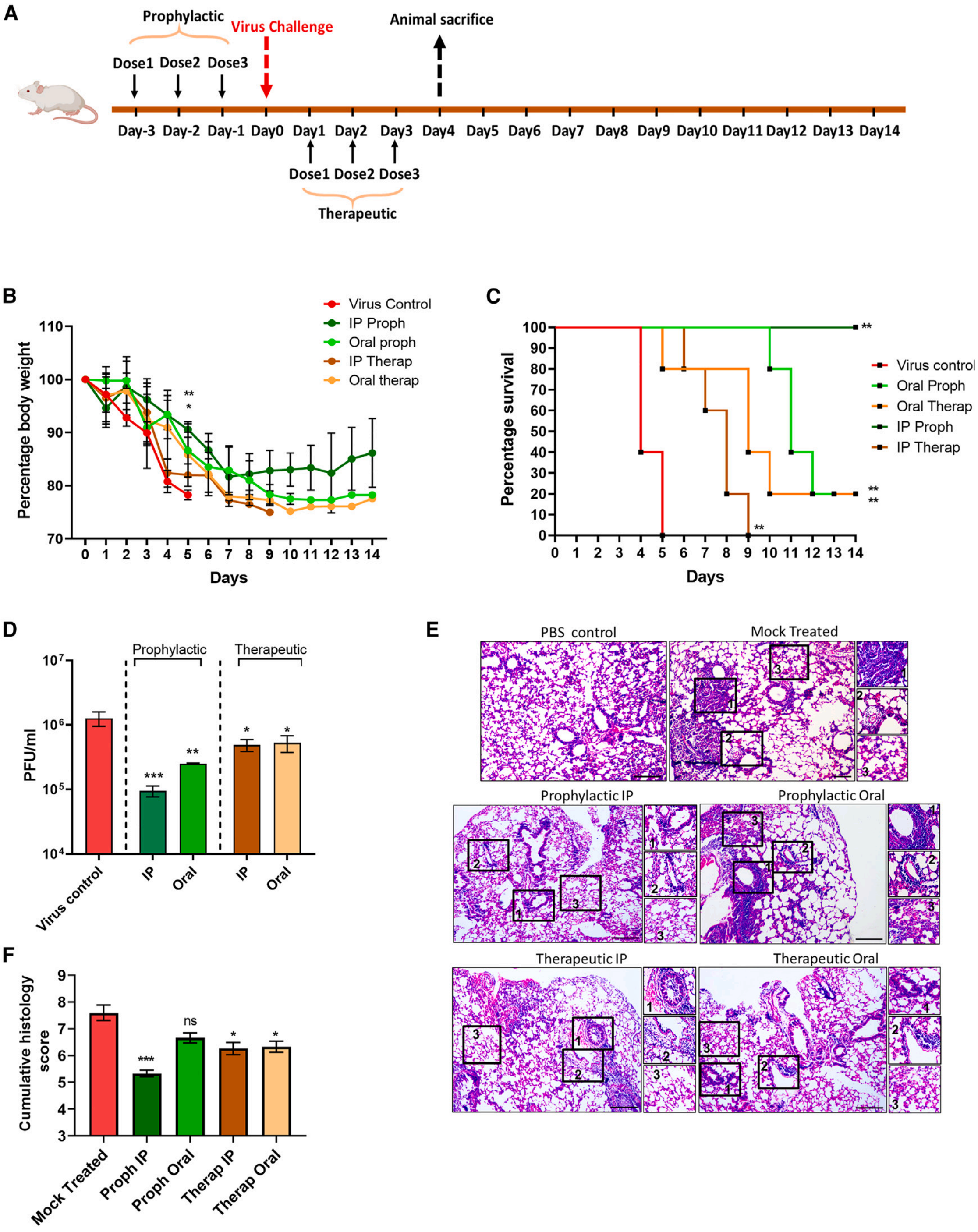

Figure 5. PA restricts IAV replication and pathogenesis *in vivo*

(A) Schematic showing virus challenge and PA treatment schedule.

(B) Body weight loss of animals upon infection and treatment monitored up to 14 dpi. Results show the mean percentage of body weight at day 0, n = 5 per group.

(C) Survival of mice (n = 5 per group) was monitored for 14 dpi for all groups.

(D) Plaque assay quantification of infectious virus titer from lungs (n = 5 per group).

(E and F) Histology images for different groups and cumulative histology scoring (n = 5 per group). Criteria used for scoring and marked in the images include (1) peribronchiolar infiltration and necrosis, (2) vascular inflammation and infiltration with inflammatory cells, and (3) alveolar infiltration. An objective histopathological scoring system was performed by a veterinarian blinded to study groups.

(legend continued on next page)

host based on toxicity assay results (Figure 4H). 1 mM PA was used for antiviral assay wherein the drug was either added at the start of the experiment or 3 h before infection of *M. smegmatis* with TM4 phage. In either case, there was no protection from TM4 mycobacteriophage infection-induced bacterial cell death (Figure 4I). This indicated that PA does not affect the entry or replication of non-enveloped bacteriophages in bacteria.

PA restricts IAV replication and pathogenesis in the murine model

With the encouraging antiviral activity of PA in cell culture, we went ahead to evaluate its *in vivo* efficacy against IAV in a BALB/c murine model. Toxicity studies in mice showed that 20 mg/kg PA was non-toxic and hence was used for further studies (Figures S5A and S5B). To test antiviral efficacy, BALB/c mice challenged with PR8 IAV were subjected to either prophylactic or therapeutic treatment of PA, administered via oral or intraperitoneal (i.p.) route (Figure 5A). While all animals in the control-infected group continuously lost weight and succumbed by day 6, the PA-treated groups showed varying levels of protection (Figures 5B and 5C). In general, prophylactic treatment and i.p. administration of PA were more effective compared with the therapeutic regimen and oral administration in preventing IAV infection-induced body weight loss and mortality, with 100% survival in the i.p. prophylactic group (Figures 5B and 5C). The antiviral effect of PA was also evident in the reduced infectious virus load in animal lungs, with a more significant effect in the prophylactic groups (Figure 5D). Furthermore, histopathological examination of lung tissue from infected animals showed significantly reduced pathology in PA-treated groups (Figures 5E and 5F).

PA has been reported to exhibit immunomodulatory effects *in vivo*.[24,25] To examine this effect in IAV challenge studies, we used flow cytometry to quantify tissue-resident T cells in the mice lung homogenates (Figures S5C and S5D). We observed a 50% increase in $CD4^+$ T cells in uninfected mice that received only PA. Moreover, the i.p. prophylactic group showed a 40% increase in $CD4^+$ T cells over virus control (Figure S5D). Quantification of macrophage inflammatory protein-1 α (MIP-1α) RNA levels in mice lungs by qRT-PCR revealed overall increased levels in i.p. treatment groups. The i.p. prophylactic group showed a significant ~40-fold increase in MIP-1α expression levels compared with the PBS control group (Figure S5E). Overall, both i.p. and oral treatment with PA displayed promising *in vivo* antiviral effects against IAV, with better outcomes in the prophylactic compared with the therapeutic regimen. The increased $CD4^+$ T cell population observed in PA-treated animals points toward additional *in vivo* effects of the drug, potentially contributing to its antiviral activity.

PA mitigates SARS-CoV-2 replication and pathogenesis in the Syrian hamster model

Next, we tested the antiviral efficacy of PA against SARS-CoV-2 in the Syrian golden hamster model using an experimental plan tested previously by our group (Figure 6A).[26] We observed that i.p. administration of a non-toxic dose of 20 mg/kg PA (Figure S6A) prophylactically and therapeutically reduced vRNA load in the lungs of infected animals 3 dpi by ~3 and ~1 orders of magnitude, respectively (Figure 6B). This was concomitant with a significant reduction in inflamed lung mass and prevention of body weight loss in PA-treated groups (Figures 6C and 6D). Similar effects were observed upon oral administration of PA (Figures 6E–6G). Histopathology analysis of lung tissue sections showed reduced pathology, shown by a marked reduction in alveolar edema and cellular infiltration of infected lungs in the PA-treated animal groups (Figures 6H and 6I). Next, we characterized the pharmacokinetics of PA in hamsters upon oral administration of the drug in either single or multiple dosages. A robust analytical method was developed with a limit of detection (LOD) of 10 ng/mL in plasma and 50 ng/mL in lung homogenates. No endogenous PA was detectable in the blank plasma and lung samples (Figures S6B and S6C). Oral administration of 20 mg/kg PA showed rapid systemic absorption, and a maximum concentration (Cmax) of 3.37 μg/mL was achieved in plasma within 30 min and was detectable up to 24 h (Figure S6D). The drug was cleared faster in the lungs and was detectable at 30 min and 1 and 2 h but not at 6 and 24 h (Figure S6E). However, upon 3 repeat doses, PA could be detected even at 24 h post-treatment at a concentration of 100 ng/gm in the lungs, indicating drug accumulation in the lungs (Figure S6E). The best outcome was observed during prophylactic treatment. Overall, these results confirm PA's antiviral activity against SARS-CoV-2 in the animal model, and oral pharmacokinetics suggest the feasibility of developing an effective oral therapeutic to combat COVID-19.

DISCUSSION

Human viruses primarily use either receptor-mediated endocytosis or viral cellular membrane fusion, or both, to enter the host cells.[27] The cellular factors involved in these host-pathogen interactions are attractive targets to develop broad-spectrum viral entry inhibitors,[7] which are less likely to be affected by viral resistance.[28] PA is a bidentate chelating ligand, which in humans is known to aid in the absorption of zinc and other trace elements from the intestine.[29] PA has been reported to exhibit antiviral activity against HIV-1, HSV-2,[30] and chikungunya virus[31]; however, the common underlying MoA remained unclear. Recently PA was shown to interfere with endocytic maturation,[10] which we hypothesized could provide broad-spectrum antiviral activity. To this end, we tested PA against IAV, SARS-CoV-2, and a panel of other enveloped viruses and found it to be effective *in vitro*. MoA studies showed that PA exerts an antiviral effect only when added before viral infection, indicating viral entry as its target. All these viruses have different genomic organizations and modes of replication; however, a common feature is the presence of the host-derived viral membrane. Also, IAV and flaviviruses require receptor-mediated endocytosis, but HSV-1 and

For (B), one-way ANOVA with Dunnett's test was done comparing against the virus control group on day 5. Data shown are from 1 experiment. Statistics are shown for i.p. prophylactic ($p = 0.0014$) and oral prophylactic ($p = 0.0252$). For (C), log rank (Mantel-Cox) was performed. For (D), one-way ANOVA with Dunnett's test was done comparing against the virus control group. Kruskal-Wallis multiple comparisons test was used in (F). *$p < 0.05$; **$p < 0.01$; ***$p < 0.001$. ns, non-significant. Scale bar, 200μm. Error bars represent mean ± SE for (F). In all other data, error bars represent mean ± SD.

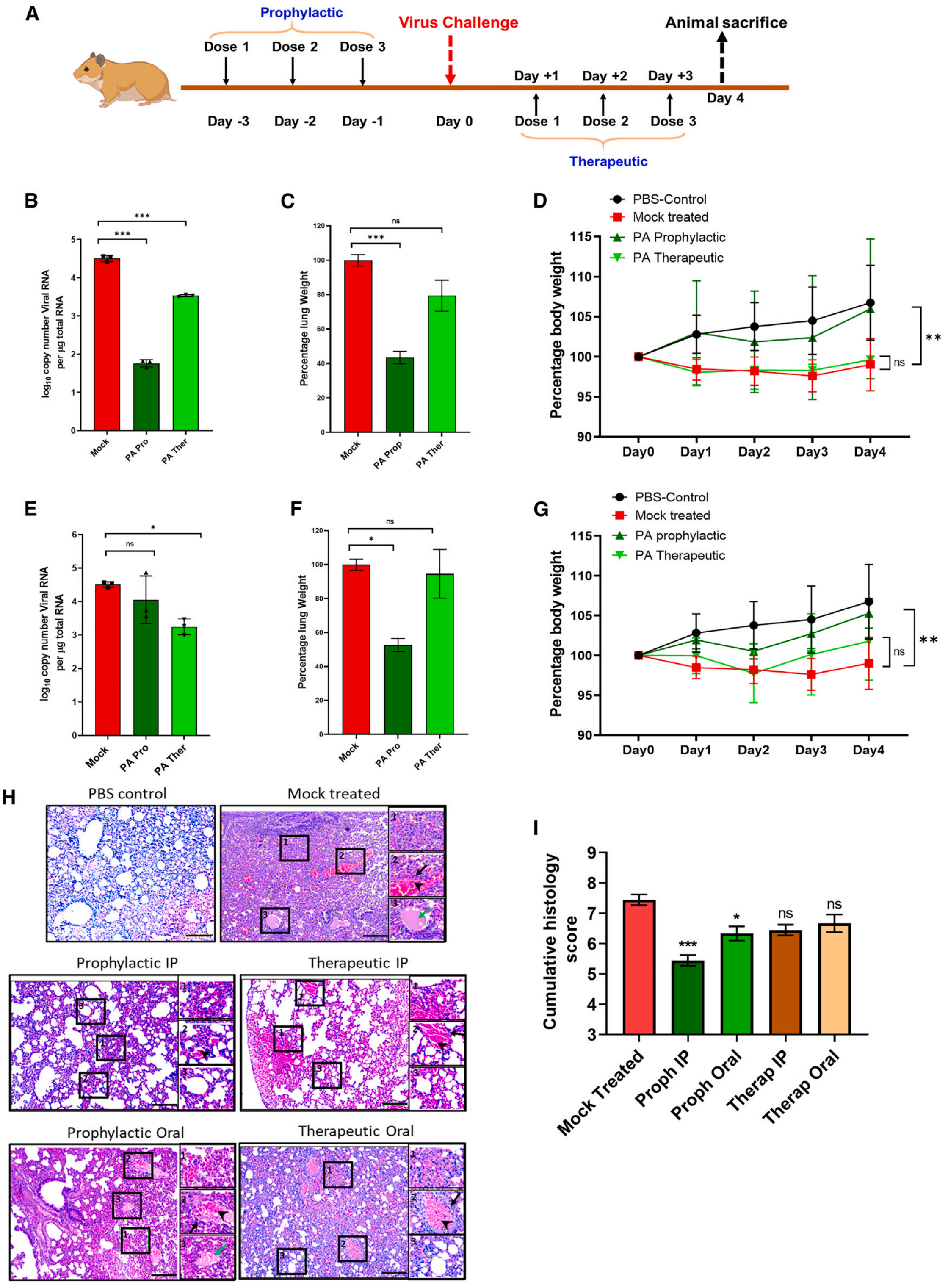

Figure 6. PA mitigates SARS-CoV-2 replication and pathogenesis *in vivo*

(A) Schematic showing virus challenge and treatment schedule.

(B–D) Dataset for PA administration via i.p. route showing (B) lung vRNA copy number (n = 3 per group), (C) total lung weight (n = 3 per group), and (D) body weight of animals up to 4 dpi (n = 4 per group).

(legend continued on next page)

SARS-CoV-2 primarily enter cells via direct viral-cellular membrane fusion,[32] suggesting that PA action may be directed on the viral membrane. For IAV, PA inhibited fusion between the viral and cellular endocytic membranes at pH 7.4 and at the cellular PM at pH 5. Also, we did not find any evidence that PA influences virus binding to cells. Notably, we observed more potent *in vitro* antiviral action of PA against SARS-CoV-2 compared with IAV. Also, PA mitigated SARS-CoV-2 more significantly in cells expressing both ACE2-TMPRSS2 receptors, indicating better inhibition of viral-cell membrane fusion than viral-endosomal membrane fusion. Exogenously added PA will likely be more effective against viral-cell membrane fusion (during SARS-CoV-2 entry) compared with less accessible viral-endocytic membrane fusion (during IAV entry) sites.

We also examined the effect of PA on endosomal positioning in PA-treated cells and found these vesicles to be dispersed away from the perinuclear region. This aligns with previous studies and adds to PA's antiviral mode of action against viruses that depend on cellular endocytosis for entry.[10] Non-enveloped viruses also often use receptor-mediated endocytosis for entry and may be impacted by PA treatment. However, our experiments revealed no effect on the entry of non-enveloped viruses like CSV, rotavirus, and Ad5. We observed the limited antiviral effect of PA on AAV6 infection, which may be due to its effect on cellular endocytosis. Membrane fusion and endocytosis are features of viral entry into eukaryotic cells. As expected, PA did not exhibit any antiviral effect on bacteriophage infection in *M. smegmatis*. This suggests that the antiviral action of PA may have evolved in higher organisms, primarily against enveloped viruses, which enter the cell via viral-cellular membrane fusion. Examination of PA-treated IAV virions by TEM revealed disruption in viral membrane integrity, which may be responsible for impaired viral-cellular membrane fusion. When we tested the effects of PA on cellular membrane integrity, we did not observe any apparent damage, however, the infectivity of PA-treated SARS-CoV-2 virions could not be restored upon washing off the PA through ultracentrifugation. It is plausible that the cellular membrane can self-repair, but the viral membrane cannot recover from PA-induced damage. Antivirals based on similar principles have been reported before.[33]

More importantly, PA treatment could restrict IAV and SARS-CoV-2 infection and pathogenesis in pre-clinical animal models. The best outcome was observed upon administration of PA prophylactically, which is consistent with its effects on early events of viral infection. We tested PA at 20 mg/kg body weight in animal studies; however, it was non-toxic up to 100 mg/kg (equivalent to 8 mg/kg in humans; conversions as per https://www.fda.gov/media/72309/download). PA has been reported to exhibit synergistic activity with interferon γ (IFN-γ) in promoting the induction of cytotoxic macrophages in mice.[24] It has also been reported to have macrophage-activating properties and is associated with MIP-1α production.[25,34,35] We observed increased levels of MIP1-α RNA in IAV-infected mice lungs treated i.p. with PA. MIP1-α is an important mediator of inflammatory reactions[36] and can regulate T cell trafficking during immune responses *in vivo*.[37,38] Interestingly, in our study, PA treatment of normal healthy and IAV-infected BALB/c mice significantly increased the CD4-positive T cell population in the lungs. The detection of PA in hamster lungs even at 24 h post-treatment indicates some accumulation of the drug in lungs, which in turn could be contributing to the recruitment of the immune effector cells. These immunomodulatory properties of PA may further enhance its antiviral activity *in vivo*. Overall, this study establishes PA as a broad-spectrum inhibitor of enveloped viral entry with promising preclinical efficacy against major respiratory pandemic viruses such as SARS-CoV-2 and IAV.

Limitations of the study

The current chemical form of PA demonstrates antiviral activity in the mM range *in vitro* and showcases limited *in vivo* bioavailability. Further exploration of its structure-activity relationship and its chemical modifications will be required to improve efficacy and pharmacokinetics. More detailed studies with larger animal numbers are required to demonstrate the *in vivo* antiviral activity against SARS-CoV-2 in a prophylactic setting. It would be interesting to follow up the findings in this study using long-COVID models. Additionally, PA, which is a natural metabolite, was exogenously administered in all experimental procedures. The metabolic pathways that regulate endogenous PA production and their contribution to antiviral immunity need further investigation.

STAR★METHODS

Detailed methods are provided in the online version of this paper and include the following:

- KEY RESOURCES TABLE
- RESOURCE AVAILABILITY
  - Lead contact
  - Materials availability
  - Data and code availability
- EXPERIMENTAL MODEL AND SUBJECT DETAILS
  - Ethics statement
  - Animal models
  - Cell lines
  - Virus stock and propagation
  - Bacterial strains

(E–G) Corresponding data for oral administration of PA. Bodyweight data are presented as the mean percentage of bodyweight measured at day 0 (n = 4 per group).
(H and I) Histology images for all groups and clinical scoring, which was done based on the following criteria labeled within inset images: (1) alveolar edema, (2) vascular and perivascular infiltration, and (3) alveolar thickening and infiltration. Black arrows indicate vascular infiltration, arrowheads show perivascular infiltration, and green arrows show alveolar edema (n = 4 per group). An objective histopathological scoring system was performed by a veterinarian blinded to study groups.
Data shown are from 1 experiment. One-way ANOVA with Dunnett's multiple comparisons was performed. *$p < 0.05$; **$p < 0.01$. Kruskal-Wallis multiple comparisons test was used in (I). Scale bar, 200 μm. Error bars represent mean ± SE for (I). In all other data, error bars represent mean ± SD.

- METHOD DETAILS
  - SARS-CoV-2 multicycle infection
  - Quantification of viral load by qRT-PCR
  - IAV multicycle infection
  - IAV plaque assay
  - Reporter virus infection
  - Flavivirus infection and plaque assay
  - Plaque assay
  - Influenza virus infection studies in mice model
  - Quantitative analysis using mice lung homogenates
  - SARS-CoV-2 infection in hamsters
  - Histopathology
  - Pharmacokinetics of PA in hamsters
  - Immunofluorescence assay
  - Western Blot
  - IAV time of addition assay
  - SARS-CoV-2 time of addition assay
  - Generation of HEK293T cells stable expressing ACE2 and TMPRSS2
  - Pseudotyped SARS-CoV-2 particle production and transduction
  - Influenza polymerase assay
  - Virus binding assay
  - Influenza virus membrane fusion assay
  - Characterizing effects of PA on viral-cellular membrane fusion
  - Testing effects of PA on cell membrane integrity
  - Characterizing effects of PA on endocytosis
  - Test of reversibility of PA effect on viral infectivity
  - Adeno associated virus - 6 production and infection
  - Adenovirus 5 infection
  - Coxsackievirus B3 infection
  - Rotavirus infection
  - Drug cytotoxicity assay
  - Transmission electron microscopy
  - Mycobacteria and TM4 bacteriophage experiments
- QUANTIFICATION AND STATISTICAL ANALYSIS

SUPPLEMENTAL INFORMATION

Supplemental information can be found online at https://doi.org/10.1016/j.xcrm.2023.101127.

ACKNOWLEDGMENTS

We thank Sushma Krishnan from the Electron Microscopy Facility and Dr. Dhanusha Gowda, Central Animal Facility of IISc, for their help in TEM imaging and histopathology scoring. We are thankful to Prof. Adolfo Garcia-Sastre, Prof. Mathew Evans, Prof. Benhur Lee (Microbiology, Icahn School of Medicine at Mount Sinai, NY, USA), Prof. Andrea Gamarnik, (Fundación Instituto Leloir-CONICET, Buenos Aires, Argentina), and Prof. David Leib (Geisel School of Medicine at Dartmouth, NH, USA) for providing different virus strains. This work was funded by the DBT-Wellcome Trust India Alliance (IA/I/18/1/503613), DBT-BIRAC (BT/CS0007/CS/02/20), the Crypto Relief Fund, L & T Trust, the DST-FIST program, the Institute of Eminence Fund, Ministry of Education, and the DBT-IISc partnership program (phase II). R.N. is supported by the DBT-BIRAC grant to the S.T. Lab. M.S., R.Y., and O.K. acknowledge support by the MHRD-IISc fellowship. S.K. and A.K. acknowledge support from Prime Minister's Research Fellowship (PMRF). A.B. acknowledges support from Kishore Vaigyanik Protsahan Yojana (KVPY) fellowship. Bacteriophage work in the R.A. laboratory was supported by the Bill & Melinda Gates Foundation (OPP1210498).

AUTHOR CONTRIBUTIONS

Conceptualization, S.T.; methodology, R.N., R.R., A.B., M.S., R.Y., O.K., S.J., P.R.S., S.K., A.K., C.M.J., and P.R.; data curation and formal analysis, R.N., R.R., A.B., S.J., and P.R.S.; resources, S.T., R.A., V.S., S.D., D.C.R., R.K.S., R.S., and S.N.; validation and visualization, R.N. and A.B.; writing – review & editing, R.N., S.T., A.B., V.S.., S.D., D.C.R., and R.A.; funding acquisition, project administration, and supervision, S.T.

DECLARATION OF INTERESTS

The Indian Institute of Science has filed patents for the use of PA and its derivatives as broad-spectrum antiviral and listed S.T., R.N., A.B., and R.R. as inventors.

Received: March 23, 2022
Revised: February 6, 2023
Accepted: June 23, 2023
Published: July 17, 2023

REFERENCES

1. Meganck, R.M., and Baric, R.S. (2021). Developing therapeutic approaches for twenty-first-century emerging infectious viral diseases. Nat. Med. *27*, 401–410.
2. Sanjuán, R., and Domingo-Calapa, P. (2016). Mechanisms of viral mutation. Cell. Mol. Life Sci. *73*, 4433–4448.
3. Iketani, S., Mohri, H., Culbertson, B., Hong, S.J., Duan, Y., Luck, M.I., Annavajhala, M.K., Guo, Y., Sheng, Z., Uhlemann, A.C., et al. (2023). Multiple pathways for SARS-CoV-2 resistance to nirmatrelvir. Nature *613*, 558–564.
4. Sheu, T.G., Fry, A.M., Garten, R.J., Deyde, V.M., Shwe, T., Bullion, L., Peebles, P.J., Li, Y., Klimov, A.I., and Gubareva, L.V. (2011). Dual resistance to adamantanes and oseltamivir among seasonal influenza A(H1N1) viruses: 2008-2010. J. Infect. Dis. *203*, 13–17.
5. Lou, Z., Sun, Y., and Rao, Z. (2014). Current progress in antiviral strategies. Trends Pharmacol. Sci. *35*, 86–102.
6. Song, J.M., and Seong, B.L. (2010). Viral membranes: an emerging antiviral target for enveloped viruses? Expert Rev. Anti Infect. Ther. *8*, 635–638.
7. Mazzon, M., and Marsh, M. (2019). Targeting viral entry as a strategy for broad-spectrum antivirals. F1000Res. *8*. F1000 Faculty Rev-1628.
8. Melillo, G., Bosco, M.C., Musso, T., and Varesio, L. (1996). Immunobiology of picolinic acid. Adv. Exp. Med. Biol. *398*, 135–141.
9. Grant, R.S., Coggan, S.E., and Smythe, G.A. (2009). The physiological action of picolinic Acid in the human brain. Int. J. Tryptophan Res. *2*, 71–79.
10. Kim, S.T., Lee, Y.J., Tasaki, T., Mun, S.R., Hwang, J., Kang, M.J., Ganipisetti, S., Yi, E.C., Kim, B.Y., and Kwon, Y.T. (2018). The N-recognin UBR4 of the N-end rule pathway is targeted to and required for the biogenesis of the early endosome. J. Cell Sci. *131*, jcs217646.
11. Mercer, J., Schelhaas, M., and Helenius, A. (2010). Virus entry by endocytosis. Annu. Rev. Biochem. *79*, 803–833.
12. Zupin, L., Fontana, F., Clemente, L., Ruscio, M., Ricci, G., and Crovella, S. (2022). Effect of Short Time of SARS-CoV-2 Infection in Caco-2 Cells. Viruses *14*, 704.
13. Aoki-Utsubo, C., Chen, M., and Hotta, H. (2018). Time-of-addition and Temperature-shift Assays to Determine Particular Step(s) in the Viral Life Cycle that is Blocked by Antiviral Substance(s). Bio. Protoc. *8*, e2830.
14. Icho, S., Rujas, E., Muthuraman, K., Tam, J., Liang, H., Landreth, S., Liao, M., Falzarano, D., Julien, J.P., and Melnyk, R.A. (2022). Dual Inhibition of Vacuolar-ATPase and TMPRSS2 Is Required for Complete Blockade of

CellPress
OPEN ACCESS

SARS-CoV-2 Entry into Cells. Antimicrob. Agents Chemother. *66*, e0043922.

15. Crawford, K.H.D., Eguia, R., Dingens, A.S., Loes, A.N., Malone, K.D., Wolf, C.R., Chu, H.Y., Tortorici, M.A., Veesler, D., Murphy, M., et al. (2020). Protocol and reagents for pseudotyping lentiviral particles with SARS-CoV-2 spike protein for neutralization assays. Viruses *12*, 513.
16. Matlin, K.S., Reggio, H., Helenius, A., and Simons, K. (1981). Infectious entry pathway of influenza virus in a canine kidney cell line. J. Cell Biol. *91*, 601–613.
17. Hoffmann, A.B., Mazelier, M., Léger, P., and Lozach, P.Y. (2018). Deciphering Virus Entry with Fluorescently Labeled Viral Particles. Methods Mol. Biol. *1836*, 159–183.
18. Yoshimura, A., and Ohnishi, S. (1984). Uncoating of influenza virus in endosomes. J. Virol. *51*, 497–504.
19. Luo, M. (2012). Influenza virus entry. Adv. Exp. Med. Biol. *726*, 201–221.
20. Sieczkarski, S.B., and Whittaker, G.R. (2003). Differential requirements of Rab5 and Rab7 for endocytosis of influenza and other enveloped viruses. Traffic *4*, 333–343.
21. To, E.E., Vlahos, R., Luong, R., Halls, M.L., Reading, P.C., King, P.T., Chan, C., Drummond, G.R., Sobey, C.G., Broughton, B.R.S., et al. (2017). Endosomal NOX2 oxidase exacerbates virus pathogenicity and is a target for antiviral therapy. Nat. Commun. *8*, 69.
22. Wu, W.T., Panté, N., Gandini, A., Aubry, N., Massoudi, M., and Antaki, J.F. (2016). Vimentin plays a role in the release of the influenza A viral genome from endosomes. Virology *20*, 41–52.
23. Kumar, N., Taily, I.M., Singh, C., Kumar, S., Rajmani, R.S., Chakraborty, D., Sharma, A., Singh, P., Thakur, K.G., Varadarajan, R., et al. (2023). Identification of diphenylurea derivatives as novel endocytosis inhibitors that demonstrate broad-spectrum activity against SARS-CoV-2 and influenza A virus both in vitro and in vivo. PLoS pathogens *19*, e1011358. https://doi.org/10.1371/journal.ppat.1011358.
24. Ruffmann, R., Welker, R.D., Saito, T., Chirigos, M.A., and Varesio, L. (1984). In Vivo Activation of Macrophages but not Natural Killer Cells by Picolinic Acid (Pla). J. Immunopharm. *6*, 291–304.
25. Bosco, M.C., Rapisarda, A., Reffo, G., Massazza, S., Pastorino, S., and Varesio, L. (2003). Macrophage activating properties of the tryptophan catabolite picolinic acid. Adv. Exp. Med. Biol. *527*, 55–65.
26. Biji, A., Khatun, O., Swaraj, S., Narayan, R., Rajmani, R.S., Sardar, R., Satish, D., Mehta, S., Bindhu, H., Jeevan, M., et al. (2021). Identification of COVID-19 prognostic markers and therapeutic targets through meta-analysis and validation of Omics data from nasopharyngeal samples. EBioMedicine *70*, 103525.
27. Marsh, M., and Helenius, A. (2006). Virus entry: open sesame. Cell *124*, 729–740.
28. Adalja, A., and Inglesby, T. (2019). Broad-Spectrum Antiviral Agents: A Crucial Pandemic Tool. Expert Rev. Anti Infect. Ther. *17*, 467–470.
29. Evans, G.W., and Johnson, P.E. (1980). Characterization and quantitation of a zinc-binding ligand in human milk. Pediatr. Res. *14*, 876–880.
30. Fernandez-Pol, J.A., Klos, D.J., and Hamilton, P.D. (2001). Antiviral, cytotoxic and apoptotic activities of picolinic acid on human immunodeficiency virus-1 and human herpes simplex virus-2 infected cells. Anticancer Res. *21*, 3773–3776.
31. Sharma, R., Fatma, B., Saha, A., Bajpai, S., Sistla, S., Dash, P.K., Parida, M., Kumar, P., and Tomar, S. (2016). Inhibition of chikungunya virus by picolinate that targets viral capsid protein. Virology *498*, 265–276.
32. Shang, J., Wan, Y., Luo, C., Ye, G., Geng, Q., Auerbach, A., and Li, F. (2020). Cell entry mechanisms of SARS-CoV-2. Proc. Natl. Acad. Sci. USA *117*, 11727–11734.
33. Wolf, M.C., Freiberg, A.N., Zhang, T., Akyol-Ataman, Z., Grock, A., Hong, P.W., Li, J., Watson, N.F., Fang, A.Q., Aguilar, H.C., et al. (2010). A broad-spectrum antiviral targeting entry of enveloped viruses. Proc. Natl. Acad. Sci. USA *107*, 3157–3162.
34. Taub, D.D., Conlon, K., Lloyd, A.R., Oppenheim, J.J., and Kelvin, D.J. (1993). Preferential migration of activated CD4+ and CD8+ T cells in response to MIP-1 alpha and MIP-1 beta *260*, 355–358.
35. Bosco, M.C., Rapisarda, A., Massazza, S., Melillo, G., Young, H., and Varesio, L. (2000). The tryptophan catabolite picolinic acid selectively induces the chemokines macrophage inflammatory protein-1 alpha and -1 beta in macrophages. J. Immunol. *164*, 3283–3291.
36. DiPietro, L.A., Burdick, M., Low, Q.E., Kunkel, S.L., and Strieter, R.M. (1998). MIP-1alpha as a critical macrophage chemoattractant in murine wound repair. J. Clin. Invest. *101*, 1693–1698.
37. Siveke, J.T., and Hamann, A. (1998). T helper 1 and T helper 2 cells respond differentially to chemokines. J. Immunol. *160*, 550–554.
38. Taub, D.D., Conlon, K., Lloyd, A.R., Oppenheim, J.J., and Kelvin, D.J. (1993). Preferential migration of activated CD4+ and CD8+ T cells in response to MIP-1 alpha and MIP-1 beta. Science *260*, 355–358.
39. Tripathi, S., Pohl, M.O., Zhou, Y., Rodriguez-Frandsen, A., Wang, G., Stein, D.A., Moulton, H.M., DeJesus, P., Che, J., Mulder, L.C.F., et al. (2015). Meta- and Orthogonal Integration of Influenza "OMICs" Data Defines a Role for UBR4 in Virus Budding. Cell Host Microbe *18*, 723–735.
40. Uchil, P.D., and Satchidanandam, V. (2003). Architecture of the flaviviral replication complex. Protease, nuclease, and detergents reveal encasement within double-layered membrane compartments. J. Biol. Chem. *278*, 24388–24398.
41. Krishna, V.D., Rangappa, M., and Satchidanandam, V. (2009). Virus-specific cytolytic antibodies to nonstructural protein 1 of Japanese encephalitis virus effect reduction of virus output from infected cells. J. Virol. *83*, 4766–4777.
42. Tripathi, S., Balasubramaniam, V.R.M.T., Brown, J.A., Mena, I., Grant, A., Bardina, S.V., Maringer, K., Schwarz, M.C., Maestre, A.M., Sourisseau, M., et al. (2017). A novel Zika virus mouse model reveals strain specific differences in virus pathogenesis and host inflammatory immune responses. PLoS Pathog. *13*, e1006258.
43. Samsa, M.M., Mondotte, J.A., Caramelo, J.J., and Gamarnik, A.V. (2012). Uncoupling cis-Acting RNA elements from coding sequences revealed a requirement of the N-terminal region of dengue virus capsid protein in virus particle formation. J. Virol. *86*, 1046–1058.
44. Schwarz, M.C., Sourisseau, M., Espino, M.M., Gray, E.S., Chambers, M.T., Tortorella, D., and Evans, M.J. (2016). Rescue of the 1947 Zika virus prototype strain with a cytomegalovirus promoter-driven cDNA clone. mSphere *1*, e00246002466-16.
45. Beaty, S.M., Park, A., Won, S.T., Hong, P., Lyons, M., Vigant, F., Freiberg, A.N., tenOever, B.R., Duprex, W.P., and Lee, B. (2017). Efficient and robust Paramyxoviridae reverse genetics systems. mSphere *2*, 003766-16.
46. Summers, B.C., and Leib, D.A. (2002). Herpes simplex virus type 1 origins of DNA replication play no role in the regulation of flanking promoters. J. Virol. *76*, 7020–7029.
47. Dhillon, P., and Rao, C.D. (2018). Rotavirus induces formation of remodeled stress granules and P bodies and their sequestration in viroplasms to promote progeny virus production. J. Virol. *92*, e01363013633-18.
48. van Ooij, M.J.M., Polacek, C., Glaudemans, D.H.R.F., Kuijpers, J., van Kuppeveld, F.J.M., Andino, R., Agol, V.I., and Melchers, W.J.G. (2006). Polyadenylation of genomic RNA and initiation of antigenomic RNA in a positive-strand RNA virus are controlled by the same cis-element. Nucleic Acids Res. *34*, 2953–2965.
49. Bortz, E., Westera, L., Maamary, J., Steel, J., Albrecht, R.A., Manicassamy, B., Chase, G., Martínez-Sobrido, L., Schwemmle, M., and García-Sastre, A. (2011). Host-and strain-specific regulation of influenza virus polymerase activity by interacting cellular proteins. mBio *2*, e00151001511-11.
50. Martin-Sancho, L., Tripathi, S., Rodriguez-Frandsen, A., Pache, L., Sanchez-Aparicio, M., McGregor, M.J., Haas, K.M., Swaney, D.L., Nguyen, T.T., Mamede, J.I., et al. (2021). Restriction factor compendium for

influenza A virus reveals a mechanism for evasion of autophagy. Nat. Microbiol. *6*, 1319–1333.

51. Schindelin, J., Arganda-Carreras, I., Frise, E., Kaynig, V., Longair, M., Pietzsch, T., Preibisch, S., Rueden, C., Saalfeld, S., Schmid, B., et al. (2012). Fiji: an open-source platform for biological-image analysis. Nat. Methods *9*, 676–682.

52. Case, J.B., Bailey, A.L., Kim, A.S., Chen, R.E., and Diamond, M.S. (2020). Growth, detection, quantification, and inactivation of SARS-CoV-2. Virology *548*, 39–48.

53. Gaush, C.R., and Smith, T.F. (1968). Replication and plaque assay of influenza virus in an established line of canine kidney cells. Appl. Microbiol. *16*, 588–594.

54. Bajpai, U., Mehta, A.K., Eniyan, K., Sinha, A., Ray, A., Virdi, S., Ahmad, S., Shah, A., Arora, D., Marwaha, D., et al. (2018). Isolation and characterization of bacteriophages from India, with lytic activity against. Can. J. Microbiol. *64*, 483–491.

55. Kalapala, Y.C., Sharma, P.R., and Agarwal, R. (2020). Antimycobacterial Potential of Mycobacteriophage Under Disease-Mimicking Conditions. Front. Microbiol. *11*, 583661.

56. Rodriguez, L., Nogales, A., and Martínez-Sobrido, L. (2017). Influenza A Virus Studies in a Mouse Model of Infection. J. Vis. Exp.

57. Chan, J.F.W., Zhang, A.J., Yuan, S., Poon, V.K.M., Chan, C.C.S., Lee, A.C.Y., Chan, W.M., Fan, Z., Tsoi, H.W., Wen, L., et al. (2020). Simulation of the Clinical and Pathological Manifestations of Coronavirus Disease 2019 (COVID-19) in a Golden Syrian Hamster Model: Implications for Disease Pathogenesis and Transmissibility. Clin. Infect. Dis. *71*, 2428–2446.

58. Crawford, K.H.D., Eguia, R., Dingens, A.S., Loes, A.N., Malone, K.D., Wolf, C.R., Chu, H.Y., Tortorici, M.A., Veesler, D., Murphy, M., et al. (2020). Protocol and Reagents for Pseudotyping Lentiviral Particles with SARS-CoV-2 Spike Protein for Neutralization Assays. Viruses *12*, 513.

59. Banerjee, I., Miyake, Y., Nobs, S.P., Schneider, C., Horvath, P., Kopf, M., Matthias, P., Helenius, A., and Yamauchi, Y. (2014). Influenza A virus uses the aggresome processing machinery for host cell entry *346*, 473–477.

60. Negrini, M., Wang, G., Heuer, A., Björklund, T., and Davidsson, M. (2020). AAV Production Everywhere: A Simple, Fast, and Reliable Protocol for In-house AAV Vector Production Based on Chloroform Extraction. Curr. Protoc. Neurosci. *93*, e103.

CellPress
OPEN ACCESS

STAR★METHODS

KEY RESOURCES TABLE

REAGENT or RESOURCE	SOURCE	IDENTIFIER
**Antibodies**		
Anti-mouse Influenza virus NP (HT103)	Center for Therapeutic Antibody Development (CTAD), Icahn School of Medicine at Mount Sinai (ISMMS), New York, USA	HT103
EEA1 (C45B10) Rabbit mAb	Cell Signaling	Cat#3288; RRID:AB_2096811
Goat anti-Mouse IgG (H + L) Cross-Adsorbed Secondary Antibody, Alexa Fluor 488	Invitrogen	Cat# A-11001; RRID:AB_2534069
Goat anti-Mouse IgG (H + L) Cross-Adsorbed Secondary Antibody, Alexa Fluor™ 568	Invitrogen	Cat# A-11004; RRID:AB_2534072
Donkey anti-Rabbit IgG (H + L) Highly Cross-Adsorbed Secondary Antibody, Alexa Fluor™ 568	Invitrogen	Cat# A10042; RRID:AB_2534017
Goat Anti-Mouse IgG - H&L Polyclonal Antibody, HRP Conjugated	Abcam	Cat# ab6789; RRID:AB_955439
Goat Anti-Rabbit IgG - H&L Polyclonal antibody, HRP Conjugated	Abcam	Cat# ab6721; RRID:AB_955447
SARS-CoV-2 (2019-nCoV) Nucleoprotein/ NP Antibody, Rabbit MAb	Sino Biological	Cat# 40143-R019; RRID:AB_2827973
SARS-CoV/SARS-CoV-2 (COVID-19) spike antibody [1A9]	GeneTex	Cat# GTX632604; RRID:AB_2864418
Polyclonal Anti-SARS-Related Coronavirus 2 Spike Glycoprotein (IgG, Rabbit),	BEI Resources, NIAID, NIH	NR-52947
Zika virus Envelope protein antibody	Genetex	Cat#GTX133314; RRID:AB_2747413
Mouse mAb to beta Actin [AC-15] (HRP)	abcam	Cat#ab49900; RRID:AB_867494
Anti-Mouse IgG (whole molecule)–Peroxidase antibody produced in goat	Sigma Aldrich	Cat#A4416; RRID:AB_258167
CD3e Monoclonal Antibody (145-2C11), APC-eFluor™ 780	Invitrogen	Cat#47-0031-82; RRID:AB_11149861
CD4 Monoclonal Antibody (RM4-5), FITC	Invitrogen	Cat#11-0042-82; RRID:AB_464896
CD8a Monoclonal Antibody (53-6.7), Super Bright™ 780	Invitrogen	Cat# 78-0081-82; RRID:AB_2722976
**Bacterial and virus strains**		
Mycobacterium smegmatis MC^2 155	Gift from Deepak Saini, Indian Institute of Science	N/A
SARS-CoV-2 (Isolate Hong Kong/ VM20001061/2020, NIAID, NIH)	BEI Resources, NIAID, NIH	NR-52282
Isolate hCoV-19/Japan/TY7-503/ 2021 (Brazil P.1)	BEI Resources, NIAID, NIH	NR-54982
Isolate hCoV-19/USA/PHC658/2021 (Lineage B.1.617.2)	BEI Resources, NIAID, NIH	NR-55611
Isolate hCoV-19/England/204820464/ 2020 (Lineage B.1.1.7)	BEI Resources, NIAID, NIH	NR-54000
Isolate hCoV-19/USA/MD-HP01542/2021 (Lineage B.1.351)	BEI Resources, NIAID, NIH	NR-55282
A/Puerto Rico/8/1934 (PR8)	Kind Gift from Prof. Adolfo Garcia-Sastre (ISMMS, NY)	Tripathi et al.[39]

(*Continued on next page*)

Continued

REAGENT or RESOURCE	SOURCE	IDENTIFIER
A/California/04/2009 H1N1 (Cal/09)	Kind Gift from Prof. Adolfo Garcia-Sastre (ISMMS, NY)	Tripathi et al.[39]
Viet Nam/1203/04 H5N1 (HALo)	Kind Gift from Prof. Adolfo Garcia-Sastre (ISMMS, NY)	Tripathi et al.[39]
West Nile virus (WNV) Strain E101	Kind Gift from Prof. Vijaya S. (Microbiology & Cell Biology, Indian Institute of Science, India)	Uchil and Satchidanandam[40]
Japanese Encephalitis Virus clinical strain P20778-GIII	Kind Gift from Prof. Vijaya S. (Microbiology & Cell Biology, Indian Institute of Science, India)	Krishna et al.[41]
Zika Virus (Cambodia strain)	Kind Gift from Prof. Adolfo Garcia-Sastre (ISMMS, NY)	Tripathi et al.[42]
IAV expressing Gaussia luciferase (NS1 Luc)	Kind Gift from Prof. Adolfo Garcia-Sastre (ISMMS, NY)	Tripathi et al.[42]
Dengue virus expressing renilla luciferase (DENV Luc).	Kind gift from Prof. Andrea Gamarnik (Fundación Instituto Leloir-CONICET, Buenos Aires, Argentina),	Samsa et al.[43]
Zika virus expressing renilla luciferase.	Kind Gift from Prof. Matthew J. Evans (ISMMS, NY)	Schwarz et al.[44]
Human parainfluenza-3 virus expressing renilla luciferase (HPIV-3 Luc).	Kind Gift from Prof. Benhur Lee (ISMMS, NY)	Beaty et al.[45]
Herpes Simplex Virus 1 expressing renilla luciferase.	Kind Gift from Prof. David Lieb, Geisel School of Medicine at Dartmouth, NH, USA	Summers and Leib[46]
Adenovirus Serotype 5, Clone Ad5-CMV-hACE2/RSV-eGFP, Recombinant Expressing Human ACE2	BEI Resources, NIAID, NIH	NR-52390
Coxsackie virus B3	Kind gift from Prof. Frank van Kuppeveld	N/A
Rhesus monkey rotavirus (RRV)	Kind gift from Durga Rao, SRM University	Dhillon and Rao[47]
TM4 mycobacteriophage	Kind gift from Rachit Agrawal, Indian Institute of Science Bajpai et al., 2018	N/A
**Chemicals, peptides, and recombinant proteins**		
Dulbecco's modified Eagle Medium	Gibco	Cat#12100046
Opti-MEM Reduced Serum Medium	Gibco	Cat#31985070
Minimum Essential Medium	Gibco	Cat#61100053
Fetal Bovine Serum, heat inactivated	Gibco	Cat#16140071
Penicillin-Streptomycin-Amphotericin B	MP Biomedicals	Cat#ICN1674049
GlutaMAX^{TM}	Gibco	Cat#35050-061
RPMI 1640	Gibco	Cat#11875085
Trypsin From Bovine Pancreas (TPCK Treated)	Sigma Aldrich	Cat#T1426
DNase I	New England Biolabs	Cat#M0303S
Poly-L-lysine	Sigma Aldrich	Cat#P9155
Polybrene	Merck	TR-1003-G
TRIzol^{TM} Reagent	Thermo Fisher	Cat#15596018
Phosphate Buffered Saline (10x)	MP Biomedicals	Cat#162528
4x Laemmli Sample Buffer	Bio-Rad	Cat#1610747
Xylazine	Indian Immunologicals Ltd.	Cat#21
Ketamine	Bharat Parenterals Limited	N/A

(*Continued on next page*)

Continued

REAGENT or RESOURCE	SOURCE	IDENTIFIER
2-Picolinic acid	Sigma Aldrich	Cat#P42800
Chloroquine diphosphate salt	Sigma Aldrich	Cat#C6628
Ammonium chloride	Fisher Scientific	Cat#21405
Carboxymethylcellulose sodium salt	Merck	Cat#C4888
Octadecyl Rhodamine B Chloride (R18)	Invitrogen	Cat#O246
DAPI for nucleic acid staining	Sigma Aldrich	Cat#D9542
ProLong^{TM} Diamond Antifade Mountant	Invitrogen	P36970
Lipofectamine 2000 transfection reagent	Invitrogen	Cat#11668019
Wheat Germ Agglutinin, Alexa Fluor^{TM} 488 Conjugate	Invitrogen	Cat#W11261
Transferrin Alexa Fluor 647 Conjugate	Invitrogen	Cat#T23366
DNase I (RNase-free)	New England BioLabs	Cat# M0303S
RNase A, DNase and protease-free	Thermo Scientific	Cat#EN0531
SalI-HF	New England BioLabs	Cat#R3138S
Carbon Type-B, 300 mesh, Copper	Ted Pella	Cat#0813
Uranyl Acetate	Ted Pella	Cat#19481
Oxoid Agar	Oxoid Limited	Cat#LP0028
DEAE-Dextran hydrochloride	Sigma Aldrich	Cat#D9885
Magnesium chloride anhydrous	HiMedia	Cat#MB237
Polyethylene glycol Mol Wt. 8000	Sigma	Cat#P-2109
Chloroform	Qualigens	Cat#Q12305
Luria broth	HiMedia	Cat#M575
Middlebrook 7H10 agar	Sigma Aldrich	Cat#M0303
ADC growth supplement	HiMedia	Cat#FD019
Collagenase type I	Merck	Cat#SCR103
Thiazolyl Blue Tetrazolium Bromide	Sigma-Aldrich	Cat#M2128
Puromycin	Gibco	Cat#A1113-03
**Critical commercial assays**		
AgPath-ID^{TM} One-Step RT-PCR kit	Applied Biosystems	Cat#AM1005
PowerUpTM SYBRTM Green Master Mix	Applied BiosystemsTM	Cat#A25742
alamarBlue^{TM} Cell Viability Reagent	Invitrogen	Cat#DAL1025
Clarity Western ECL Substrate	Bio-Rad	Cat#1705061
Firefly luciferase assay kit	Promega	Cat#E4550
Dual-Luciferase Reporter Assay System	Promega	Cat#E1980
LIVE/DEAD^{TM} Fixable Aqua Dead Cell Stain Kit	Invitrogen	Cat#L34966
Prime ScriptTM RT Reagent Kit with gDNA Eraser	Takara-Bio	RR047A
**Experimental models: Cell lines**		
HEK293T-ACE2	BEI Resources, NIAID, NIH	NR-52511
Vero E6	ATCC	CRL-1586
HEK293T	NCCS, Pune, India	N/A
Caco-2	NCCS, Pune, India	N/A
A549	NCCS, Pune, India	N/A
MDCK	NCCS, Pune, India	N/A
HeLa	ATCC	CCL-2
**Experimental models: Organisms/strains**		
Syrian Golden Hamster	Biogen laboratory animal facility	N/A
BALB/c mice	Central Animal Facility, Indian Institute of Science	N/A

(*Continued on next page*)

**Continued**		
REAGENT or RESOURCE	SOURCE	IDENTIFIER
Oligonucleotides		
SARS-CoV-2 N1 Primers	Merck	N/A
SARS-CoV-2 N1 Probe	Merck	N/A
18S rRNA	GCC Biotech	N/A
MIP1-alpha	GCC Biotech	N/A
Recombinant DNA		
Vector pHDM Containing the SARS-Related Coronavirus 2, Wuhan-Hu-1 Spike Glycoprotein,	BEI Resources, NIAID, NIH	NR-52514
SARS-Related Coronavirus 2, Wuhan-Hu-1 Spike D614G-Pseudotyped Lentiviral Kit Lentiviral Backbone, Luc2; ZsGreen	BEI Resources, NIAID, NIH	NR-52516
SARS-Related Coronavirus 2, Wuhan-Hu-1 Spike D614G-Pseudotyped Lentiviral Kit Helper plasmid, Gag; pol	BEI Resources, NIAID, NIH	NR-52517
SARS-Related Coronavirus 2, Wuhan-Hu-1 Spike D614G-Pseudotyped Lentiviral Kit Helper plasmid, Tat1b	BEI Resources, NIAID, NIH	NR-52518
SARS-Related Coronavirus 2, Wuhan-Hu-1 Spike D614G-Pseudotyped Lentiviral Kit Helper plasmid, Rev1b	BEI Resources, NIAID, NIH	NR-52519
pAdDeltaF6	Gift from James M. Wilson	Addgene plasmid # 112867; RRID:Addgene_112867
pRepCap6	Gift from David Russell	Addgene plasmid # 110770; RRID:Addgene_110770
pAAV-CAG-GFP	Gift from Edward Boyden	Addgene plasmid # 37825; RRID:Addgene_37825
pCB3/T7	Kind gift from Prof. Frank van Kuppeveld	van Ooij et al.[48]
IAV Mini replicon Plasmids (PA, PB1, PB2, NP, NP-Luciferase)	Kind Gift from prof. Adolfo Garcia-Sastre (ISMMS, NY)	Bortz et al.[49]
pRLTK	Promega	E2231
pWPI-IRES-Puro-Ak-ACE2-TMPRSS2	Gift from Sonja Best	Addgene plasmid # 154987; RRID:Addgene_154987)
gag-pol	Kind Gift from prof. Adolfo Garcia-Sastre (ISMMS, NY)	Martin-Sancho et al.[50]
VSV-G	Kind Gift from prof. Adolfo Garcia-Sastre (ISMMS, NY)	Martin-Sancho et al.[50]
Software and algorithms		
BioRender	BioRender	https://biorender.com/
ImageJ/Fiji	Schindelin et al.[51]	https://imagej.nih.gov/ij/
QuantStudio Design and Analysis Software v1.5.1	Applied Biosystems	https://www.thermofisher.com/in/en/home/global/forms/life-science/quantstudio-3-5-software.html
CytExpert Acquisition and Analysis Software Version 2.3	Beckman Coulter	https://www.mybeckman.in/flow-cytometry/instruments/cytoflex/software
GraphPad Prism 8.4.3	GraphPad Software	https://www.graphpad.com/scientific-%20software/prism/
Magellan	Software V7.1 SP1	https://lifesciences.tecan.com/software-magellan
LAS X	Version 3.7.6.25997	https://www.leica-microsystems.com/products/microscope-software/p/leica-las-x-ls/

(*Continued on next page*)

Continued

REAGENT or RESOURCE	SOURCE	IDENTIFIER
Other		
Transmission Electron Microscope	Thermo Scientific	Talos L120C
CytoFLEX flow cytometer	Beckman Coulter	A00-1-1102
Confocal multiphoton microscope	Zeiss	Zeiss LSM 880
Confocal microscope	Leica	Leica SP8 Falcon (FLIM, FCS)
Ultracentrifuge	Beckman Coulter	L8-70M
ChemiDoc^{TM} MP Imaging System	BIO RAD	12003154
Quantstudio 5 Real-Time PCR Instrument 384-well Block)	Applied Biosystems	A28135
TECAN Infinite 200-PRO multiplex reader.	TECAN	https://lifesciences.tecan.com/plate_readers/infinite_200_pro
7205 UV/Visible scanning spectrophotometer	Jenway	http://www.jenway.com/product.asp?dsl=9171
Tecan Spark multi-mode plate reader	Tecan	https://lifesciences.tecan.com/multimode-plate-reader
Formvar/carbon-covered 300 mesh copper grid	Ted Pella	Cat#01753-F
PVDF membrane	Immobilon-P; Merck	Cat#IPVH00010
Amicon Ultra-0.5 Centrifugal Filter Unit	Merck-Millipore	Cat#UFC510008
Whatman® UNIFLO® 25 syringe filters, pore size 0.45 μm	Sigma Aldrich	Cat#9913-2504
Whatman® qualitative filter paper, Grade 1	Sigma Aldrich	Cat#WHA1001125

RESOURCE AVAILABILITY

Lead contact

Further information and requests for resources and reagents should be directed to and will be fulfilled by the lead contact, Shashank Tripathi (shashankt@iisc.ac.in).

Materials availability

This study did not generate new reagents.

Data and code availability

- All data reported in this paper will be shared by the lead contact upon request.
- This paper does not report any original code.
- Any additional information required to reanalyze the data reported in this paper is available from the lead contact upon request.

EXPERIMENTAL MODEL AND SUBJECT DETAILS

Ethics statement

This study was conducted in compliance with institutional biosafety guidelines, (IBSC/IISc/ST/17/2020; IBSC/IISc/ST/18/2021), following the Indian Council of Medical Research and Department of Biotechnology recommendations. All experiments involving animals were reviewed and approved by the Institutional Animal Ethics Committee (Ref: IAEC/IISc/ST/784/2020) at the Indian Institute of Science and Foundation for Neglected Disease Research (ref. 2082/Po/Rc/S/19/CPCSEA). All SARS-CoV-2 experiments and IAV animal work were performed in a Viral Biosafety level-3 facility. The experiments were performed according to CPCSEA (The Committee for Control and Supervision of Experiments on Animals) guidelines.

Animal models

For IAV infection experiments, 4-6 weeks-old female BALB/c mice (Central Animal Facility, Indian Institute of Science, Bengaluru, India) were used. Animal experiments involving SARS-CoV-2 infection were performed on 10-12 week-old mixed-gender Syrian golden hamsters (Biogen Laboratory Animal Facility Bengaluru, India) with male and female hamsters housed separately. All animals were housed in groups of four in individually ventilated cages maintained at 23 ± 1°C temperature and 50 ± 5% relative humidity, given access to standard pellet feed and water *ad libitum* and maintained on a 12-h day/night light cycle at the Viral Biosafety

level-3 facility, Indian Institute of Science. All animals were monitored daily during the experiment. An overdose of Ketamine (Bharat Parenterals Limited) and Xylazine (Indian Immunologicals Ltd) was used to sacrifice animals upon completion of the experiment.

Cell lines

The following cell lines were used in this study: HEK293T (NCCS, Pune, India), HEK293T cells expressing human ACE2 (HEK293T-ACE2) (NR-52511, BEI Resources, NIAID, NIH), HEK293T cells stably expressing ACE2 and TMPRSS2 (HEK293T-ACE2-TMPRSS2) generated by transducing HEK293T cells with lentiviruses expressing the receptors, Vero E6 (CRL-1586, ATCC), Madin-Darby Canine Kidney (NCCS, Pune, India); A549 (NCCS, Pune, India), Caco-2 (NCCS, Pune, India), HeLa (ATCC, CCL2), A549 (NCCS, Pune). All cell lines were cultured in complete Dulbecco's modified Eagle medium (12100-038, Gibco) with 10% HI-FBS (16140-071, Gibco), 100 IU/mL Penicillin and 100 μg/mL Streptomycin (15140122, Gibco) supplemented with GlutaMAX (35050-061, Gibco).

Virus stock and propagation

The following SARS-CoV-2 isolates were procured from BEI Resources, NIAID, NIH: Isolate Hong Kong/VM20001061/2020, NR-52282; Isolate hCoV-19/England/204820464/2020 (Lineage B.1.1.7; Alpha variant), NR-54000; Isolate hCoV-19/USA/MD-HP01542/2021 (Lineage B.1.351 South Africa; Beta variant), NR-55282; Isolate hCoV-19/USA/PHC658/2021 (Lineage B.1.617.2; Delta Variant), NR-55611; Isolate hCoV-19/Japan/TY7-503/2021 (Brazil P.1 Gamma variant), NR-54982. All these viruses were propagated and titrated by plaque assay in Vero E6 cells as described before.[52] Influenza A virus (IAV) strains namely A/Puerto Rico/8/1934 H1N1 (PR8) and A/California/04/2009 H1N1 (Cal/09) and Viet Nam/1203/04 H5N1 (HALo) were propagated in 11-day old embryonated chicken eggs and titrated by plaque assay in MDCK cells.[53] The reporter viruses used in this study include IAV expressing Gaussia luciferase (NS1 Luc)[39,53]; Japanese Encephalitis Virus (JEV) clinical strain P20778 was propagated and titrated in BHK-21 cells. The reporter viruses used in this study include IAV expressing Gaussia luciferase (NS1 Luc)[39]; Dengue virus (DENV Luc), Zika Luc, Human parainfluenza virus (HPIV-3 Luc), and Herpes Simplex Virus (HSV-1 Luc) expressing renilla luciferase, were provided from different laboratories (details in resource table). Adenovirus Serotype 5, Clone Ad5-CMV-hACE2/RSV-eGFP, Recombinant Expressing Human ACE2 was procured from BEI resources (Catalog No. NR-52390). West Nile virus (WNV) Strain E101 was a kind Gift from Prof. Vijaya S (Indian Institute of Science), and Zika Virus (Cambodia strain) was a kind Gift from Prof. Adolfo Garcia-Sastre (ISMMS, NY). Coxsackie virus B3 (CVB3) was a kind gift from Prof. Frank van Kuppeveld. Rhesus monkey rotavirus (RRV) strain was a kind gift from Prof. Durga Rao. C, SRM University, Andhra Pradesh. TM4 mycobacteriophage[54] was amplified in *M. smegmatis* and phage enumeration was done using the soft agar overlay technique as reported by Kalapala et al.[54,55]

Bacterial strains

Primary *Mycobacterium smegmatis* (mc^2 155) (a kind gift from Prof. Deepak Saini, Indian Institute of Science) was grown in Middlebrook 7H9 broth (Merck, M0178) supplemented with Glycerol (Fisher scientific, Q24505), ADC (HiMedia, FD019), and 0.1% v/v Tween 80 (Fisher Scientific, YBP338500). A log-phase primary culture was inoculated into a secondary culture without Tween 80 and supplemented with 2 mM $CaCl_2$ (Fisher scientific, Q12135) to promote efficient infection of phages.

METHOD DETAILS

SARS-CoV-2 multicycle infection

HEK293T-ACE2, HEK 293T-ACE2-TMPRSS2, Caco-2, or Vero E6 cells in 24-well dishes were pre-treated with 0.25, 0.5, 1, and 2 mM PA for 3 h in triplicates and infected with 0.01 or 0.001 MOI of SARS-CoV-2 respectively. For infection, 100 μL of inoculum was used for 1 h adsorption with intermittent shaking every 10 min, then topped up with 400 μL medium. Infection in HEK293T-ACE2 and Vero E6 cells was done using complete DMEM and DMEM containing 2% FBS respectively. After 48 h, total RNA was extracted using TRIzol (Thermo Fisher, 15596018), triplicates for each condition were pooled together, and viral copy number was estimated by qRT PCR. Cell viability of uninfected, drug-treated cells was measured using MTT (Sigma, M2128) assay as per the manufacturer's instructions. The drug was present in the medium throughout the experiment. A similar protocol, but using only 2 mM PA, was used to test the drug against SARS-CoV-2 variants of concern Alpha, Beta, Gamma, Delta, or Omicron in HEK293T-ACE2 and Vero E6 cells.

Quantification of viral load by qRT-PCR

Cells were harvested in TRIzol as per the manufacturer's instructions. An equal amount of RNA was used to determine viral load using the AgPath-ID One-Step RT-PCR kit (Applied Biosystems, AM1005). The following primers and probes targeting the SARS-CoV-2 N-1 gene were used for amplification. Forward primer: 5′GACCCCAAAATCAGCGAAAT3' and Reverse primer: 5' TCTGGTT ACTGCCAGTTGAATCTG3′, Probe: (6-FAM/BHQ-1) ACCCCGCATTACGTTTGGTGGACC). The Ct values were used to determine viral copy numbers by generating a standard curve using the SARS-CoV-2 genomic RNA standard.

IAV multicycle infection

MDCK cells in 24-well dishes were treated with 0.25, 0.5, 1, and 2 mM PA for 3 h in triplicates, infected with 100 μL per well 0.001 MOI of PR8 in Opti-MEM reduced serum media (Gibco, 31985088) containing 1 μg/mL TPCK trypsin (Sigma Aldrich, T1426) in triplicates.

For Cal/09 virus, a single dose of 2 mM PA was used for treatment. After 1 h, wells were topped up with 500 μL OptiMEM 48 hpi supernatants from each condition were pooled, centrifuged at 2000xg to remove cell debris, and used for plaque assay. In all cases, PA was present in media throughout the experiment. Uninfected cells treated with different doses of PA were used for estimation of cell viability by MTT assay.

IAV plaque assay

Dilutions (10-fold) of supernatants were prepared in Opti-MEM and 150 μL per well was used to infect confluent MDCK cells in 12-well dishes, in duplicates, for 1 h at 37°C. Virus inoculum was then removed, and cells were overlaid with 1 mL MEM (Gibco, 61100053) containing 0.6% oxoid agar (Thermo Scientific, LP0028), 1 μg/mL TPCK trypsin, 0.01% DEAE-Dextran hydrochloride (Sigma Aldrich, D9885), and 0.5% NaHCO3 (MP Biomedicals, 194553). Cells were collected at 48 hpi, fixed with 4% paraformaldehyde (PFA), and plaques were visualized by crystal violet staining.

Reporter virus infection

A549 cells in 24-well dishes were either treated with 2 mM PA for 3 h, washed and infected with reporter viruses, or the drug was added to 6 hpi. Cells were infected with 100 μL per well DMEM containing 1 μL of DENV Luc, ZIKV Luc; 0.2 μL HPIV-3 Luc or 1 μL HSV-1 Luc. Once added, the drug was present in the medium throughout the remaining duration of the experiment. After 48 h, cells were harvested for detecting luciferase expression using the Dual-Luciferase Reporter Assay System (Promega, E1980) as per the manufacturer's instructions. Luminescence measurements were taken using a TECAN Infinite 200-PRO multiplex reader.

Flavivirus infection and plaque assay

A549 cells in 24-well dishes were pre-treated for 3 h with 2 mM PA and infected with 100 μL per well DMEM containing 0.1 MOI WNV clinical strain in triplicates. After 1 h of adsorption, the wells were topped up with 400 μL DMEM. Drug (2 mM PA) was present in the media throughout the experiment. Cells were then washed with PBS and harvested for western blot analysis 48 hpi. The separated proteins were transferred onto a PVDF membrane and probed using mouse anti-Flavivirus envelope 4G2 primary antibody (Center for Therapeutic Antibody Development (CTAD), Icahn School of Medicine at Mount Sinai (ISMMS), NY), and anti-mouse-HRP conjugated secondary antibody (Abcam, ab6789. RRID: AB_955439). Actin labeling using Mouse mAb to beta Actin-HRP (Abcam, ab49900. RRID: AB_867494) was used as a loading control.

Plaque assay

Cell culture supernatants were harvested and centrifuged at 1000 x g and 10-fold dilutions were prepared in 2% PBS containing DMEM. Plaque assay was performed in confluent BHK-21 cells in 6 well plates. After 1 h adsorption, the inoculum was removed and cells were overlaid with DMEM containing 2% carboxymethylcellulose (CMC) (Merck, C4888), and 2% FBS. After 5 days, cells were fixed with 4% PFA for 30 min and stained with crystal violet.

Influenza virus infection studies in mice model

Mice were used since they are the most widely used preclinical animal model for Influenza virus research.[56] To assess toxicity, animals were treated with 20, 50, or 100 mg/kg PA by either IP or Oral routes. The body weight and general health of animals were measured every day for up to 14 days post-treatment. Infection/treatment groups were divided into two, one group receiving 20 mg/kg PA prophylactically and the other, therapeutically. This corresponds to a human equivalent dose of 20 mg/kg (Mouse) x 0.08 (the conversion factor) = 1.6 mg/kg (conversions as per https://www.fda.gov/media/72309/download). For infection, mice under IP anesthesia with Ketamine (90 mg/kg) and Xylazine (4.5 mg/kg) were challenged intranasally with 1000 PFU of PR8 IAV virus in 40 μL PBS. Two dosage regimens and routes of drug administration were followed for the treatment of animals. The prophylactic treatment used the administration of 20 mg/kg/day PA via oral or IP route during −3, −2, and −1 day before infection and therapeutic dosage (oral/IP) involved administering 20 mg/kg/day PA during 1, 2 and 3-dpi. One-half of the animals were sacrificed at 4 dpi and lungs were collected for plaque assay, histology, and flow cytometry analysis. For the remaining animals, total body weight and survival were recorded until the end of the experiment at 14 dpi. For plaque assay, lung samples were collected in DMEM containing 0.3% bovine serum albumin (Sigma, A7906), homogenized, and centrifuged at 5000xg for 10 min at 4°C to pellet tissue debris. The supernatant was used for plaque assay as described previously.

Quantitative analysis using mice lung homogenates

Flow cytometry analysis

During organ collection, mice lung tissues were washed in PBS to remove residual blood and stored in RPMI 1640 containing 10% FBS. Enzyme solution for tissue homogenization was prepared in PBS containing 0.5% BSA, by adding 5 mg/mL collagenase-type 1 (Merck, Cat#SCR103) and 1 mg/mL DNase I (NEB, M0303S). Lung tissue samples were added to enzyme solution, homogenized manually using glass slides, and incubated at 37°C for 30 min to facilitate tissue disruption. Samples were then centrifuged at 1000 rpm for 5 min at 4°C, resuspended in RBC lysis buffer (0.15M NH_4Cl, 1mM $NaHCO_3$, 0.1mM EDTA), and incubated for 5 min at RT. After washing once with RPMI containing 0.5% BSA, the samples were passed through a 70 μm Cell Strainer (Corning, 431751), centrifuged, resuspended in FBS containing 10% DMSO and stored at −80°C until used for FACS analysis. An aliquot of

each sample containing ~10^6 cells was loaded into a 1.5 mL centrifuge tube, centrifuged and the supernatant was removed by decanting. To each tube 200 μL PBS containing 2 mM EDTA was added, briefly vortexed, and incubated at RT for 15 min to homogenize the cell suspension. Samples were then centrifuged at 3000 rpm for 5 min at 4°C, the supernatant was discarded and stained with a Live/Dead cell stain kit (Invitrogen, L34966) as per the manufacturer's instructions. Samples were washed once with FACS buffer (PBS with 0.3% BSA) and stained for surface markers CD3 (Invitrogen, 47-0031-82), CD4 (Invitrogen, 11-0042-82), and CD8 (Invitrogen, 78-0081-82) as per manufacturer's instructions. Data acquisition was performed on a Beckman Coulter CytoFLEX flow cytometer (A00-1-1102). Automated compensation was performed using CytExpert 2.5.0.77 software with unstained and single-stained controls. For each sample, 10^5 events were obtained and analyzed by using CytExpert. The following hematopoietic lineage populations were defined: $CD3^+$, $CD4^+$, and $CD8^+$.

Quantitative real-time PCR of MIP1-alpha

One portion of the lung was homogenized and used for RNA extraction using TRIzol reagent (Thermo Fisher, 15596018) as per manufacturer's instruction. 1 μg of RNA was reverse transcribed into cDNA using Prime ScriptTM RT Reagent Kit with gDNA Eraser (Perfect Real Time - RR047A, Takara-Bio) and then diluted 5-fold with nuclease-free water (112450204, MP Biomedicals). The gene expression study was conducted using PowerUpTM SYBRTM Green Master Mix (A25742, Applied BiosystemsTM) with 18S rRNA as the internal control and appropriate primers for the genes.

18S rRNA - Fwd: GTAACCCGTTGAACCCCATT, Rev: CCATCCAATCGGTAGTAGCG.

MIP1-alpha - Fwd: CCATATGGAGCTGACACCCC, Rev: GAGCAAAGGCTGCTGGTTTC.

SARS-CoV-2 infection in hamsters

Syrian golden hamsters were used since they are currently considered the animal model of choice for the evaluation of vaccines and antivirals against SARS-CoV-2. Hamsters under intraperitoneal (IP) Ketamine (150 mg/kg) and Xylazine (10 mg/kg) anesthesia were challenged intranasally with 10^5 plaque-forming units (PFU) SARS-CoV-2 in 100 μL PBS. Two dosage regimens and routes of drug administration were followed for the treatment of animals. The prophylactic treatment used the administration of 20 mg/kg/day PA via oral or IP route during −3, −2, and −1 day before infection and therapeutic dosage (oral/IP) involved administering 20 mg/kg/day PA during 1, 2 and 3-day post-infection (dpi). This corresponds to a human equivalent dose of 20 mg/kg (Hamster) x 0.13 (the conversion factor) = 2.6 mg/kg (conversions as per https://www.fda.gov/media/72309/download). A total volume of 200 μL PA dissolved in PBS was used for both oral and IP routes of administration. The total body weight of animals was recorded every day until the end of the experiment at 4 dpi when animals were sacrificed. Total lungs were harvested, weighed, and processed for histopathological analysis. One portion was used for RNA extraction using TRIzol and subsequent viral RNA copy number estimation by qRT-PCR as described previously.

Histopathology

Lung tissue samples were fixed in 10% buffered PFA, embedded in paraffin blocks, and tissue sections of 6 μm thickness were made using a microtome. The sections were then stained with Hematoxylin and Eosin and examined by light microscopy as previously described.[57] Clinical scoring for hamster lung samples was done based on three different criteria namely: Alveolar edema; vascular and perivascular infiltration; alveolar thickening and infiltration. In the case of mice, three different clinical criteria were observed, namely: vascular infiltration, alveolar infiltration, and interstitial pneumonia. In both cases, scoring was done based on the severity on a scale of 1–4 (1-mild, 2-moderate, 3-severe, 4-very severe). Histopathology analysis and scoring were done by a trained veterinary pathologist who was blinded to treatment groups.

Pharmacokinetics of PA in hamsters

Syrian golden hamsters (10–12-week-old) were administered with 20 mg/kg PA dissolved in 0.5% hydroxypropyl methyl cellulose (HPMC), via the oral route. The drug was administered either as a single dose or with 3 multiple doses at 24-h intervals, and the pharmacokinetics of PA was evaluated by detecting levels of the drug at time points 0.5, 1, 2, 4, 6 and 24 h for plasma samples and 0.5, 1, 2, 6 and 24 h for lung samples. Waters Acquity Ultra-Performance Liquid Chromatography (UPLC) system was used for detection with the following parameters: Column: Eclipse Plus C18 (75 × 4.6 × 3.5 μm), Mobile phase A: 1% formic acid in Water, Mobile phase B: 1% formic acid in Acetonitrile, Flow: Gradient, Flow Rate: 0.250 mL/min, Inj. Volume-7μL, Run Time: 3.5 min, AS Temperature.: 15°C, Column Temperature.: 40°C, Internal Standard: Linezolid −200 ng/mL.

Immunofluorescence assay

Cells on glass coverslips were washed once with PBS (162528, MP Biomedicals), fixed with 4% PFA for 10 min, and permeabilized for 5 min with PBS containing 0.1% Triton X-100 (Sigma, T8787). Cells were then washed and incubated in a blocking buffer (PBS with 0.01% Triton X-100, 2% BSA) for 1 h. Overnight incubation with primary antibody diluted in blocking buffer was followed by washing and incubation with secondary antibody in blocking buffer containing 0.01 μg/mL DAPI (Sigma Aldrich, D9542). Finally, cells were incubated with PBS containing 50 mM NH_4Cl (Fisher Scientific, 21405) for de-quenching, washed, and the cells on coverslips were mounted on glass slides using ProLong Diamond Antifade Mountant (Invitrogen, P36961).

Western Blot

Cells were washed with 1x PBS, lysed with 1x Laemmli buffer (1610747, BIO-RAD), and heated at 95°C for 10 min. Cell lysates were then subjected to standard SDS-PAGE and separated proteins were transferred onto a PVDF membrane (IPVH00010, Immobilon-P; Merck). The membrane was incubated in a blocking buffer containing 5% Skimmed milk (Sigma Aldrich, 70166) in PBS containing 0.05% Tween 20 (Sigma-Aldrich P1379) (1xPBST) for 2 h with slow rocking at room temperature (RT). Primary antibody incubation in blocking buffer was done for 14 h at 4°C with gentle rocking, after which the membrane was washed with 1x PBST and incubated for 2 h with secondary antibody in blocking buffer at RT. After a further wash with 1x PBST, the blots were developed using Clarity Western ECL Substrate (Bio-Rad, 1705061).

IAV time of addition assay

For infection, A549 cells in 24 well dishes containing glass coverslips (or not) were washed once with warm PBS and incubated with 100 μL per well 5 MOI PR8 IAV virus diluted in OptiMEM (Gibco, 31985062). The plates were rocked intermittently to ensure an even distribution of inoculum. After 1 h of adsorption, the medium in the wells was topped up with 400 μL OptiMEM. Effects of PA on early and late events during IAV infection were studied at 3 and 9 hpi, respectively. In the former, A549 cells were first pre-treated with 2 mM PA for 3 h, media removed and infected with 5 MOI PR8 IAV in the presence of 2 mM PA. After 3 h incubation at 37°C/5% CO2, cells were either fixed with 4% PFA for IFA, or cell lysates were collected using 1X Laemmli buffer for western blot. The direct effect of PA on virus particles was studied by first treating the virus inoculum prepared in OptiMEM with 2 mM PA for 1 h at 37°C. This was then used for infection of untreated A549 cells and 3 hpi, cells were collected for IFA and western blot as above. The virus particles were not washed prior to infection and no additional PA was added to cells. The effects of PA on late events during virus infection were studied by infecting A549 cells and treating cells 6 hpi. Cells were then incubated for a further 3 h before collection. For IFA, anti-mouse Influenza virus NP (HT103) (CTAD, ISMMS, NY) and Goat anti-Mouse, Alexa Fluor 488 (Invitrogen, A1100. RRID: AB_2534069) was used as primary and secondary antibodies respectively. Images were acquired using a Leica SP8 confocal microscope. Quantification of NP-positive cells relative to the total number of DAPI-positive cells in 4 different fields was performed using ImageJ/Fiji software. Primary antibody incubation for western blot was done with Anti-mouse Influenza virus NP (HT103) and secondary antibody with Goat Anti-Mouse IgG - H&L Polyclonal Antibody, HRP conjugated (Abcam, ab6789. RRID: AB_955439).

SARS-CoV-2 time of addition assay

For infection, Vero E6, HEK293T-ACE2, or HEK293T-ACE2-TMPRSS2 cells in 24-well dishes containing glass coverslips were washed once with warm PBS and incubated with 100 μL per well 10 MOI SARS-CoV-2 diluted in DMEM containing 2% FBS (2% DMEM). The plates were rocked every 10 min to ensure an even distribution of inoculum. After 1 h of adsorption, the media in wells were topped up with 400μL 2% DMEM. Effects of PA on early and late events during SARS-CoV-2 infection were studied at 3 and 9 h time points p.i, respectively. In the former, cells were first pre-treated with 2 mM PA for 3 h, media removed, and infected with 10 MOI SARS-CoV-2 in the presence of 2 mM PA. After 3 h incubation at 37°C/5% CO2, cells were either fixed with 4% PFA for IFA, or cell lysates were collected using 1X Laemmli buffer for western blot. The direct effect of PA on virus particles was studied by first treating the virus inoculum prepared in 2% DMEM with 2 mM PA for 1 h at 37°C. This was then used for infection of untreated cells and 3 hpi, cells were collected for IFA and western blot as above. No additional PA was added to cells here. PA treatment of cells simultaneously during infection of HEK 293T-ACE2 cells (T0) was also done to test the entry effects of the drug at the time of infection. The effects of PA on late events during virus infection were studied by infecting cells and treating cells 6 hpi. Cells were then incubated for a further 3 h before collection. For IFA, SARS-CoV-2 spike (GTX632604, GeneTex. RRID: AB_2864418) and Goat anti-Mouse IgG (H + L) Cross-Adsorbed Secondary Antibody, Alexa Fluor 488 (Invitrogen, Cat# A-11001, RRID: AB_2534069) were used as primary and secondary antibodies respectively. Quantification of spike-positive cells was done using ImageJ/Fiji. Western blot analysis of viral proteins used Polyclonal Anti-SARS-Related Coronavirus 2 Spike Glycoprotein (BEI, NR-52947) and Goat Anti-Rabbit IgG - H&L Polyclonal antibody, HRP Conjugated (Abcam, ab6721. RRID: AB_955447).

Generation of HEK293T cells stable expressing ACE2 and TMPRSS2

Lentivirus preparation

HEK293T cells in a 6wp were transfected per well with 1 μg pWPI-IRES-Puro-Ak-ACE2-TMPRSS2 (Addgene #154987), 0.6 μg Gag-Pol and 0.4 μg VSVG plasmid using Lipofectamine 2000 as per manufacturer's instructions. 3 hpi, the medium was replaced with fresh complete DMEM and incubated at 37°C/5% CO2. After 48 h, the supernatant was collected, and stored at 4°C. Cells were replenished with fresh DMEM and incubated for a further 24 h before collecting supernatant again. The supernatants were then pooled together, passed through a 0.2 μm syringe filter, and stored in aliquots at −80°C before transduction.

Transduction

HEK293T cells were seeded in 6wp to reach 80% confluency after 24 h. The transduction mixture was prepared by mixing lentivirus preparation with complete DMEM in a 1:1 v//v ratio and containing 1 μg/mL polybrene (Merck, TR-1003-G). Cell culture medium from the 6wp was removed and replaced with 2mL per well of the transduction mixture. Cells were then incubated at 37°C/5% CO2 for 24 h and the medium was removed and replaced with fresh DMEM. After 12 h, a medium containing 1 μg/mL Puromycin (Gibco, A1113-03) was added and further incubated for 48 h. Once complete cell death in the un-transduced cells was observed, transduced cells were replenished with fresh DMEM without antibiotic and after a further 12 h recovery, cells were trypsinized and seeded in a T75

flask. Transduced cells were maintained in a medium containing 1 μg/mL Puromycin for up to 4 passages before being used for experiments.

Pseudotyped SARS-CoV-2 particle production and transduction

Pseudotyped particles bearing the SARS-CoV-2 spike protein were produced as reported before.[58] HEK293T-ACE2 cells were treated with 2, 1, 0.5, and 0.25 mM PA and 3 h later, transduced with 100 μL per well-pseudotyped SARS-CoV-2 particles containing 5μg/mL polybrene. The different concentrations of PA were present throughout the experiment. EDTA (1 mM) was used as a control. Post transduction (60 h), cells were washed once with PBS and processed for detecting luciferase expression using a Firefly luciferase assay kit (Promega, E4550) as per the manufacturer's instructions. Luminescence measurements were taken using a TECAN Infinite 200-PRO multiplex reader.

Influenza polymerase assay

HEK293T cells in 24-well dishes were then co-transfected with IAV Mini replicon plasmids including 50 ng each of PA, PB1, PB2; 200ng of Nucleoprotein (NP); 50ng NP-firefly Luciferase; (kind gift from Adolfo Garcia Sastre, ISMMS, NY)[49] and 100ng pRLTK as an internal control. Post 3 h transfection, cells were treated with 2 mM PA, harvested at 8 and 12 h post-treatment for detection of firefly and renilla luciferase expression using Dual-Luciferase Reporter Assay System (Promega, E1980) as per manufacturer's instructions. Luminescence readings were taken using a TECAN Infinite 200-PRO multiplex reader.

Virus binding assay

The effect of PA on virus binding was tested by incubating either cells or the virus inoculum itself with the drug. A549 cells were trypsinized to make a cell suspension and resuspended in the infection medium (0.2% BSA in DMEM) for infection. The effect of PA on cells was tested by pre-incubating cells with 2 mM PA for 3 h at 37°C/5% CO2, followed by infection. The effect of PA on virus particles was evaluated by incubating the virus inoculum with the drug for 1 h at 37°C before being used for infection. Alternatively, the PA-treated virus preparation was washed by ultracentrifugation at 30000 rpm for 2 h at 4°C to remove traces of PA from the inoculum and subsequently used for infection. In all conditions, PR8 IAV, 50 MOI was used for infection on ice for 1 h, followed by which cells were washed with ice-cold PBS to remove unbound virus particles, and fixed with 4% PFA for 10 min. Cells were then washed once with warm PBS, incubated with FACS buffer (0.3% BSA in PBS) for 30 min, washed, and incubated with mouse anti-HA antibody for 3 h at RT. Cells were again washed 3 × 5 min with PBS and incubated with anti-mouse Alexa Flour 647 secondary antibody for 1 h in the dark, washed, and resuspended in FACS buffer. FACS analysis of Alexa Flour 647 positive cells was performed using a Beckman coulter Cytoflex flow cytometer.

Influenza virus membrane fusion assay

Virus labeling

IAV particles were labeled using Octadecyl Rhodamine B Chloride (R18) (Invitrogen, O246) as reported earlier, with minor modifications. A total volume of 1 mL PR8 IAV (8×10^8 PFU/mL) was centrifuged at 10,000 x g to remove debris, R18 dye was added at 100 μM final concentration and the mixture was continuously vortexed at RT for 6 min. The virus-dye mixture was then placed on ice for 2 h, passed through a 0.4 μm syringe filter to remove unbound aggregates, and stored at −80°C.

Membrane fusion assay

The methodology was adapted from a previous report with few modifications. MDCK cells in suspension were pre-treated for 3 h with 2 mM PA or 10 μM Ammonium chloride (NH_4Cl) (Fisher scientific 21405), at 37°C. Cells were then washed with ice-cold PBS and infected with 10 MOI R18 labeled virus in infection medium (DMEM containing 0.2% BSA, pH 7.4), 1 μg/mL trypsin, and placed on ice. Post 1 h, cells were washed twice with ice-cold PBS and resuspended in an infection medium containing 2 mM PA or 10 μM NH_4Cl. For PM fusion assay, R18 labeled viruses were induced to fuse at the plasma membrane by incubating cells in a fusion medium (DMEM containing 0.2% BSA, adjusted to pH 5.0 using citrate buffer) at 37°C for 2 min, followed by washing and resuspension in cold infection medium with 2 mM PA. In both cases, cells were then quickly transferred to a pre-chilled opaque flat-bottom 96-well dish which was then placed in a TECAN Infinite 200-PRO multiplex reader pre-set at 37°C. Fluorescence intensity measurements (Ex 560/Em 590) were recorded at every 10 min interval for up to 60 min.

Characterizing effects of PA on viral-cellular membrane fusion

The protocol was adapted from previous reports, with a few modifications.[16,23,59] A549 cells were pre-treated with 2 mM PA for 3 h and infected with 10 MOI PR8 IAV on ice for 60 min. For endocytosis bypass, virus fusion at the plasma membrane was initiated by incubating cells with a fusion medium at 37°C for 2 min. Regular IAV infection via endocytosis used infection medium at pH 7.4. As a negative control, cells were allowed to fuse in the presence of 50 mM NH_4Cl at pH 7.4, which does not allow PM fusion. Cells were then quickly washed with ice-cold PBS and incubated with an infection medium containing 50 mM NH4Cl. After the fusion step, PA was not present in the infection medium. 10 hpi, cells were washed twice with warm PBS, fixed with 4% paraformaldehyde for 10 min and IFA was done to label NP using mouse anti-Influenza NP (HT103) and anti-mouse Alexa Flour 488 secondary antibody. Nuclei were labeled with DAPI. Images were acquired using a Leica SP8 confocal microscope at 63x magnification. The number of NP-positive puncta was quantified using ImageJ/Fiji.

Testing effects of PA on cell membrane integrity

A549 cells grown on glass coverslips were treated with 2 mM PA for 3h. Cells were then washed with warm PBS and fixed with 4% PFA for 10 min at RT. Cells were then incubated with PBS containing 5 μg/mL WGA-488 for 10 min at 37°C. After labeling, cells were washed twice with PBS and nuclei labeled in with 0.1 μg/mL DAPI in PBS for 5 min. Cells were washed again in PBS and the cover slips mounted were on a glass slide. A Leica SP8 Falcon confocal microscope was used to capture multiple z stacks and 3D surface projections were created using the LAS X tool.

Characterizing effects of PA on endocytosis

Immunofluorescence assay for IAV NP protein and cellular EEA-1

A549 were cells pre-treated with 2 mM PA for 3 h and infected with 10 MOI PR8 IAV on ice for 60 min. Cells were then washed twice in ice-cold PBS to remove unbound virus and moved to 37°C for another 60 min in the presence of the drug. Cells were then fixed with 4% PFA for 10 min, permeabilized, and labeled with mouse anti-IAV NP (HT103) and Rabbit anti-EEA1 (C45B10) (Cell Signaling, #3288). Secondary antibodies used were anti-mouse Alexa Fluor 488 (Invitrogen, 11001) and anti-rabbit Alexa Fluor 568 (Invitrogen, A10042). Images were acquired using a Leica SP8 confocal microscope. Las X software (Leica) was used to calculate Pearson's correlation coefficients for the analysis of colocalization between NP and EEA-1 labeled structures.

PA effects on localization of endocytic vesicles

A549 cells in a 24-well dish containing glass coverslips were pre-treated for 3 h with 2 mM PA and incubated with 100 μL/well Opti-MEM containing 25 μg/mL Transferrin 647 (Tf-647) on ice. After 1 h, cells were washed twice with warm PBS and OptiMEM containing 2 mM PA was added to wells. After 30 min chase, cells were washed and fixed with 4% PFA in PBS for 10 min. Images were acquired using a Leica SP8 confocal microscope. ImageJ/Fiji was used to draw line ROIs up to a distance of 15 μm from the nuclei and the fluorescence intensity of Tf 647 loaded vesicles was measured.

Test of reversibility of PA effect on viral infectivity

A preparation of SARS-CoV-2 particles in complete DMEM was incubated with 2 mM PA for 60 min at RT. One-half of the preparation was then resuspended in an excess of PBS and pipetted multiple (10) times. The remaining treated virus served as the control for unwashed virus preparation. The virus in PBS suspension was left at RT for 15 min and the washed virus was purified by ultracentrifugation (30000 rpm for 2 h at 4°C) through a 30% sucrose cushion prepared in NTC buffer (1 M NaCl, 0.2 M Tris-HCl pH 7.4, 50 mM CaCl2). The virus was then resuspended in complete DMEM and used for infection. Untreated virus control used in the experiment was also concentrated by the same method. Infection was done at 10 MOI and no additional PA was added. Cells were collected at 3 hpi and viral spike expression levels were analyzed by western blot using rabbit anti-spike antibody (BEI resources, NR-52947).

Adeno associated virus - 6 production and infection

AAV6 particles were produced as per a previously published protocol[60] with few modifications. Briefly, HEK293T cells were seeded in 2 X T75 flasks to reach 50–60% confluency the next day. For each flask, the following plasmids were transfected using Lipofectamine 2000 transfection reagent (Invitrogen, 11668019) as per manufacturer instructions: 17.7 μg pAdDeltaF6 (Addgene 112867), 7.9 μg pRepCap6 (Addgene 110770), and 5.9 μg pAAV-CAG-GFP (Addgene 37825). After 60 h, the cells and medium mixture were pooled and transferred to a 50mL conical tube. 3mL Chloroform (Q12305, Qualigens) was added, and vortexed gently for 5 min, and 8mL 5M NaCl was added. The tube was then centrifuged for 5 min at 3000 × g, 4°C, and the aqueous phase was transferred to a fresh tube. 10mL of 50% (v/v) PEG 8000 (Sigma, P-2109) was added, vortexed briefly, and incubated for 1 h on ice before centrifuging for 30 min at 3000 × g, 4°C. The supernatant was then discarded, the pellet re-suspended in 1.5mL HEPES (H5303, Promega), vortexed for 2 min, and the following components were added: 3.5μL of 1M $MgCl_2$ (HiMedia, MB237), 14μL DNase I (NEB, M0303S) and 1.4μL of 10 μg/μL RNase A (Thermo Scientific, EN0531). The contents were incubated for 20 min at 37°C, and an equal volume of chloroform was added to the tube, and mixed well before centrifuging for 5 min at 3000 × g. The aqueous phase was then transferred to a fresh tube, followed by which the contents were passed through a 100kDa Amicon Ultra-0.5 Centrifugal Filter Unit (Merck-Millipore, UFC510008) by centrifugation for 5 min at 14,000 × g. The column was washed twice with PBS and AAV particles were eluted into a fresh tube by centrifugation at 1000 × g for 2 min.

For infection, HEK293 in a 24-well dish was pre-treated with 2 mM PA for 3 h and infected with 100 μL complete DMEM per well containing three different volumes i.e. 2, 5, and 10 μL AAV6 particles. After 1 h, the medium was topped up with 400 μL complete DMEM. PA was present in the medium for the entire duration of the experiment. After 48 h, cells were trypsinized and re-suspended in PBS containing 3% FBS. The number of GFP-positive cells was analyzed using a Cytoflex flow cytometer and results were analyzed using CytExpert software.

Adenovirus 5 infection

For infection studies, HEK293T cells were pre-treated for 3 h with 2 mM PA and infected with 10 MOI Adenovirus Serotype 5, Clone Ad5-CMV-hACE2/RSV-eGFP (BEI Resources NR-52390) in the presence of the drug. After 24 h, cells were trypsinized, resuspended in FACS buffer, and used to quantify the total number of GFP-positive cells by flow cytometry.

Coxsackievirus B3 infection

Virus preparation

CVB3 virus was prepared as reported earlier (*34, 45*). Briefly, pCB3/T7 DNA was linearized using the SalI-HF enzyme (R3138S, NEB) and CVB3 RNA was produced by *in vitro* transcription reaction. The infectious RNA was transfected in HeLa cells and the cell culture supernatant was harvested after 48 h. The virus was amplified in one passage. For virus titer calculation, a plaque assay was performed in Vero E6 cells and plaque-forming units per milliliter were estimated.

Plaque assay and infection

Vero E6 cells were seeded in a 12-well plate at a confluency of approximately 90% and infected with serially diluted CVB3-containing cell culture supernatant and incubated at 37°C for 1 h with the gentle swirling of the medium at every 10–15 min interval. After virus adsorption, cells were washed with PBS and overlaid with a 1:1 mixture of 2X DMEM and 1.6% Low melting agarose (Sigma-Aldrich, A9414), and incubated at 37°C. After 48 h, cells were fixed using 4% paraformaldehyde for 1 h and stained with 1% crystal violet solution.

To study the early effects of PA, HeLa cells were pre-treated for 3 h, infected with 10 MOI CVB3, and collected at 3 hpi. Alternatively, cells were infected and treated simultaneously (T0) and collected after 3 h. A mixture of virus and drug incubated for 1 h (1 h PA + Virus) was used for infection to test the effects of PA on virus particles. No additional drug was added here post-infection. Expression levels of VP1 protein were detected by western blot using monoclonal mouse anti-enterovirus primary antibody Clone 5-D8/1 (Dako, M7064) and anti-mouse antibody-HRP (Sigma Aldrich, A4416).

Rotavirus infection

A working concentration of RRV was prepared by diluting the virus stock 2-fold with complete DMEM containing 2 μg/mL TPCK trypsin. The mixture was incubated at 37°C for 30 min and further diluted 2-fold in serum-free DMEM. This mixture was used to infect HEK293T cells that were pre-treated for 3 h with 2 mM PA. A volume of 100 μL per well, in a 24-well plate was used for 1 h adsorption, after which the wells were topped up with 400 μL serum-free media containing PA. After 12 h, cells were fixed with 4% PFA, immunolabeled with mouse anti VP6 antibody (kind gift from C.Durga Rao) and Alexa Fluor 568 secondary antibody (Invitrogen, A11004) for quantification of infected cells using a Beckman Coulter Cytoflex flow cytometer. IFA analysis of infected cells used mouse anti-VP6 antibody and anti-mouse Alexa Fluor 488 antibody (Invitrogen, A-11001).

Drug cytotoxicity assay

A549 cells were seeded in 96-well dishes and 24 h later treated with 0.25, 0.5, 1, 2, 4, 8, 16, and 32 mM PA in triplicates. Cells were then incubated at 37°C, 5% CO2, and cytotoxicity was measured at 48 h post-treatment using MTT assay.

Transmission electron microscopy

PR8 IAV virus of stock titer 2 × 109 PFU/mL grown in 11-day-old embryonated chicken eggs was passed through a 0.45 μm syringe filter and concentrated by ultracentrifugation at 25,000xg for 2 h at 4°C. The concentrated virus prep was then incubated with either distilled water (vehicle control) or 2 mM PA for 1 h at RT. Aliquots (2–3 μL) of the virus samples were applied to a Formvar/carbon-covered 300 mesh copper grid (Ted Pella, 01753-F) which was hydrophilized by glow discharging at 8 W for 60 s directly before use. After 2 min, the excess sample was removed using Whatman filter paper (Sigma Aldrich,1001125). 5 μL of negative stain 2% uranyl acetate (SRL Lab, 81405) was added to the grid and incubated for 40 s, after which excess stain was removed. The negative staining step was repeated 3 times and the grid was air-dried for 10 min before imaging. The grids were imaged using a Talos L120C transmission electron microscope equipped with a LaB6 electrode operating at an acceleration voltage of 120 kV. Images of the virus were recorded using a 4k Å∼ 4k Ceta CMOS camera.

Mycobacteria and TM4 bacteriophage experiments

Bacterial toxicity assay

PA stock solution of 1M concentration was prepared in sterile deionized water and diluted to obtain different concentrations (1 mM, 5 mM, 10 mM, 20 mM, and 40 mM), in a 48-well plate. A total of 2×10^5 cells of *M. smegmatis* mc2 155 were added to each of the wells. The 48-well plate was placed in a rotary shaker incubator at 37°C for 24 h. Readings were taken periodically using a Tecan Spark multi-mode plate reader at 600 nm.

Effect of drug on TM4 mycobacteriophage infection

To study the effect of the drug on TM4 phage growth and activity, 7H9 broth supplemented with ADC growth supplement (HiMedia, FD019) and Calcium chloride (Fisher Scientific Q12135) was prepared and inoculated with 100 μL of log-phase secondary bacterial culture (OD 1–2) per 5mL of the media. 1 mM of the drug was added to culture tubes at the appropriate time (either at the start of the experiment or 3 h before adding the phage in the mid-log phase). The cultures were then incubated at 37°C with rotary shaking at 180 p.m. For phage-treated samples, a 10 MOI TM4 mycobacteriophage was added at a specified interval of the mid-log phase. For optical density (OD) measurements, 100 μL of bacterial culture at various time intervals was diluted 10 times in media and pipetted several times to obtain a uniform cell suspension. Readings were taken using Jenway 7205 UV/Visible Spectrophotometer at 600 nm against a media blank.

QUANTIFICATION AND STATISTICAL ANALYSIS

All statistical analyses were performed using GraphPad Prism 8.4.3 (GraphPad Software, USA). Details about the statistical method used are mentioned in the legend section of the respective figures. In main Figures 5, 6, S5, and S6 'n' represents the number of animals per group. Error bars which indicate either SD or SEM have been mentioned in the respective figure legends. In all cases, a p value <0.05 is considered significant. Graphical illustrations were created using http://biorender.com/.

Cell Reports Medicine, Volume *4*

Supplemental information

Picolinic acid is a broad-spectrum inhibitor of enveloped virus entry that restricts SARS-CoV-2 and influenza A virus *in vivo*

Rohan Narayan, Mansi Sharma, Rajesh Yadav, Abhijith Biji, Oyahida Khatun, Sumandeep Kaur, Aditi Kanojia, Christy Margrat Joy, Raju Rajmani, Pallavi Raj Sharma, Sharumathi Jeyasankar, Priya Rani, Radha Krishan Shandil, Shridhar Narayanan, Durga Chilakalapudi Rao, Vijaya Satchidanandam, Saumitra Das, Rachit Agarwal, and Shashank Tripathi

Supplemental item titles

1. **Fig S1. *In vitro* cytotoxicity of PA and effect of control antivirals against IAV and SARS-CoV-2. Related to Figure 1**
2. **Fig S2. PA inhibits SARS-CoV-2 entry in HEK293T-ACE2 cells. Related to Figure 2**
3. **Fig S3. PA treatment does not inhibit virion binding to the host cell but impedes its endocytic movement toward the nucleus. Related to Figure 3**
4. **Fig S4. PA inhibits ph5.0-induced fusion of IAV with the plasma membrane, does not affect cell membrane architecture but has an irreversible effect on treated virion infectivity. Related to Figure 3**
5. **Fig. S5. *In vivo* toxicity profile and immunomodulatory effects of PA in BALB/c mice. Related to Figure 5**
6. **Fig. S6. *In vivo* toxicity profile of PA in Syrian golden Hamsters and pharmacokinetics of PA in animal lungs and blood plasma. Related to Figure 6**

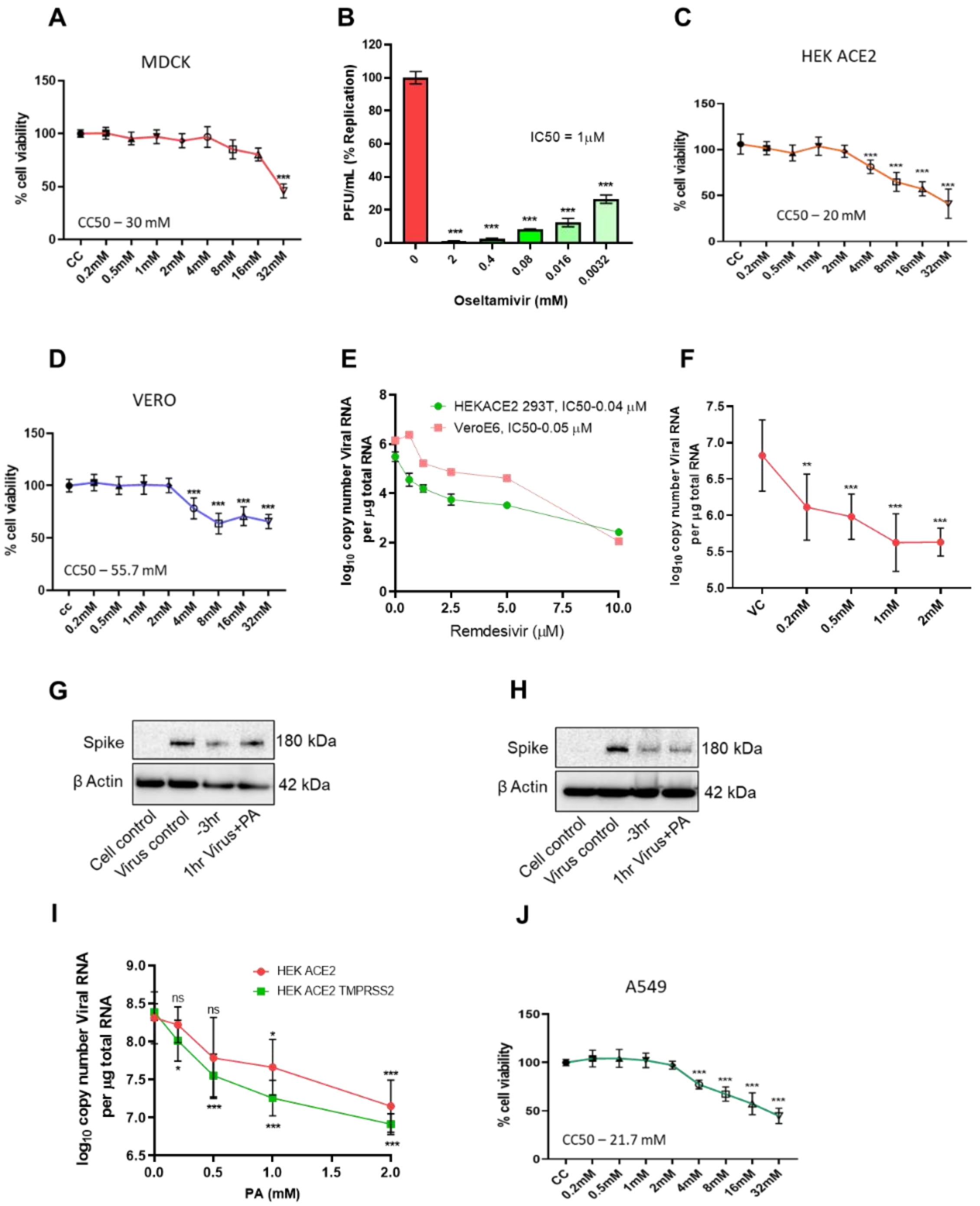

Fig S1. ***In vitro*** **cytotoxicity of PA and effect of control antivirals against IAV and SARS-CoV-2. Related to Figure 1.** **(A)** MDCK cells were treated with increasing doses of PA as indicated, and cell viability was measured by MTT assay after 48 hr**. (B)** A549 cells were pre-treated for 3 hr with different concentrations of Oseltamivir and infected with 0.001 MOI Cal/09 virus. Infectious virus from cell supernatants was measured by plaque assay. **(C-D)** HEK293T-ACE2 and Vero E6 cells were treated with increasing doses of PA as indicated, and cell viability was measured by MTT assay after 48 hr**. (E)** HEK293T-ACE2 and VeroE6 cells were pre-

treated for 3 hr with increasing concentrations of Remdesivir as indicated and infected with 0.01 MOI (HEK293T-ACE2) or 0.001 MOI (VeroE6). Total cellular RNA was collected at 48 hpi and vRNA was estimated by qRT PCR. **(F)** Caco-2 cells were pre-treated with increasing doses of PA as indicated, infected with 0.01 MOI SARS-CoV-2 and 72 hpi, and vRNA load was estimated by qRT PCR. **(G,H)** HEK293T-ACE2 and HEK293T-ACE2-TMPRSS2 cells were first pre-treated for 3 hr with 2 mM PA (-3hr), infected with 10 MOI SARS-CoV-2 in the presence of the drug, and collected 3 hpi. To test the direct effects of PA on virus particles, the virus inoculum was incubated with 2 mM PA for 1 hr at 37°C and subsequently used for infection (1 hr virus + PA). No additional PA was added here. Viral spike protein expression levels by western blot are shown for (G) HEK293T-ACE2 and (H) HEK293T-ACE2-TMPRSS2 cells. **(I)** HEK293T-ACE2 and HEK293T-ACE2-TMPRSS2 cells were pre-treated with increasing doses of PA as indicated, infected with 0.01 MOI SARS-CoV-2 and 48 hpi, vRNA load was estimated by qRT PCR. **(J)** A549 cells were treated with increasing doses of PA as indicated, and cell viability was measured by MTT assay after 48 hr**.** The data comprises 3 independent biological replicates, with datasets including 2-3 technical replicates. ***$p < 0.001$, using a two-tailed unpaired *t*-test, one-way ANOVA with Dunnett's multiple comparison test, or Brown–Forsythe and Welch ANOVA with Dunnett's T3 multiple comparison test wherever necessary. Error bars represent mean ± SD.

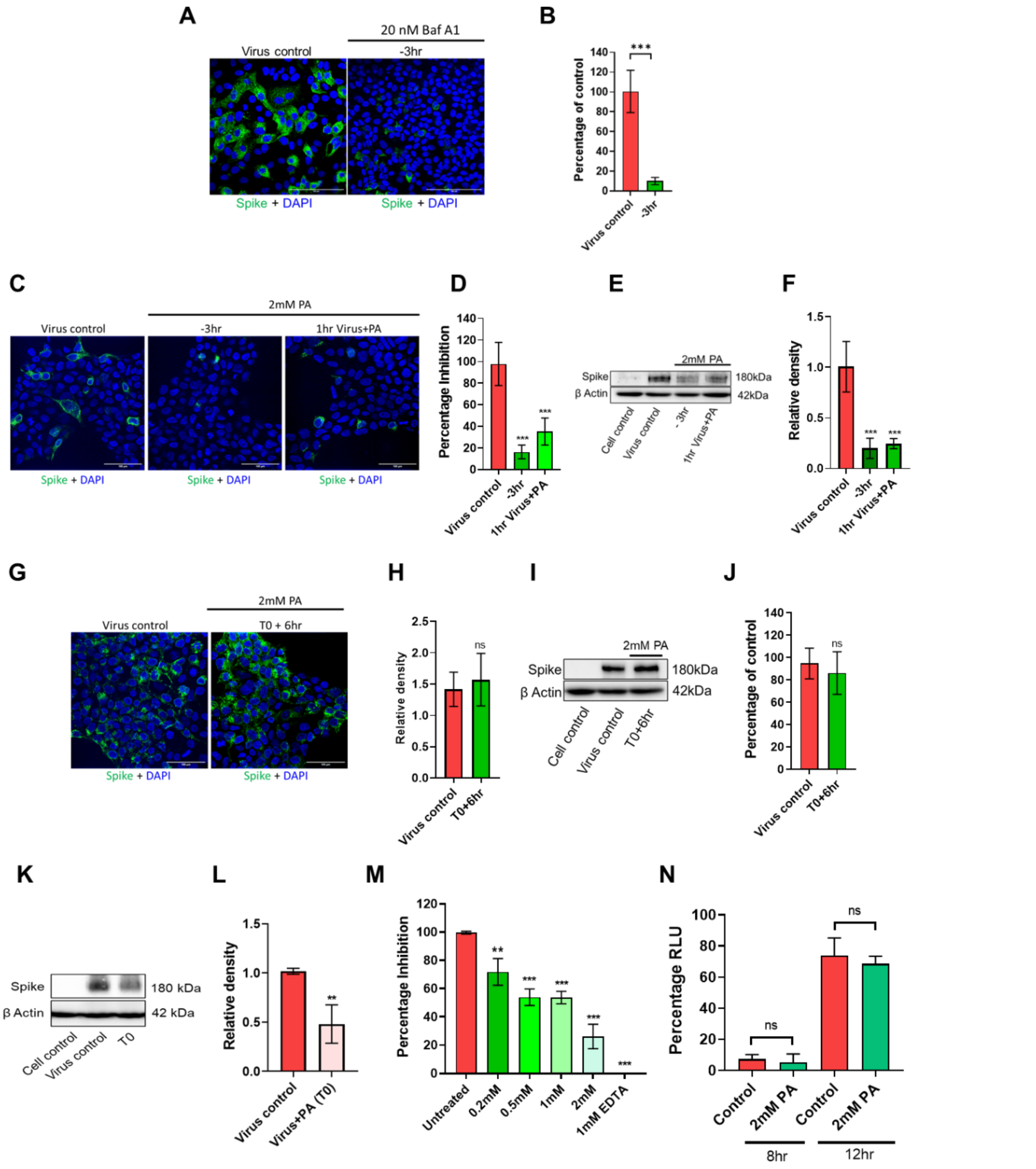

Fig S2. PA inhibits SARS-CoV-2 entry in HEK293T-ACE2 cells. Related to Figure 2. (A-B) Vero E6 cells were pre-treated for 3 hr with 20 nM Bafilomycin A1 and infected with 10 MOI SARS-CoV-2 in the presence of the drug. Cells were then collected at 3 hpi. (A) Confocal images showing viral spike positive cells in green and (B) quantification of cells in green**. (C-F)** Time of addition assay was performed in HEK293T-ACE2 cells by pre-treating cells for 3 hr with 2 mM PA (-3hr), infecting with 10 MOI SARS-CoV-2 in the presence of the drug, and collecting 3 hpi. Alternatively, the virus inoculum was incubated with 2 mM PA for 1 hr at 37°C and subsequently used for infection (1 hr virus + PA). No additional drug was added. (C) Confocal images showing

viral spike positive cells in green and (D) quantification of cells in green. (E) Spike expression levels by western blot for the above conditions and quantification of (F) band intensity. **(G-J)** Untreated HEK293T-ACE2 were first infected with 10 MOI SARS-CoV-2 and treatment with 2 mM PA was done at 6 hpi (T0+6 hr). Cells were then collected 3 hr post addition of the drug. Confocal images with quantification of spike-positive cells and corresponding western blot data are shown in (G,H) and (I,J), respectively. Scale bar = 100μm. (**K, L**) HEK 293T-ACE2 cells were infected with 10 MOI SARS-CoV-2 and treated simultaneously with 2 mM PA. Cell lysates were collected 3 hpi and viral spike protein expression was estimated by (K) western blot analysis and (L) relative band intensity quantified using ImageJ/Fiji. **(M)** HEK293T-ACE2 cells were pre-treated with increasing doses of PA or 1 mM EDTA as indicated, infected with SARS-CoV-2 spike pseudotyped particles, and luciferase expression levels measured 60 hpi **(N)** HEK293T cells were transfected with IAV mini replicon plasmids PA, PB1, PB2, NP, and NP-firefly luc, with pRLTK control. PA (2 mM) was added 3 hr post-transfection, cells were collected 8 and 12 hr later, and luciferase expression was estimated. Data are from 3 independent experiments. $^{**}p < 0.01$, ns – nonsignificant using a two-tailed unpaired *t*-test or one-way ANOVA with Dunnett's multiple comparison test wherever necessary. Error bars represent mean ± SD.

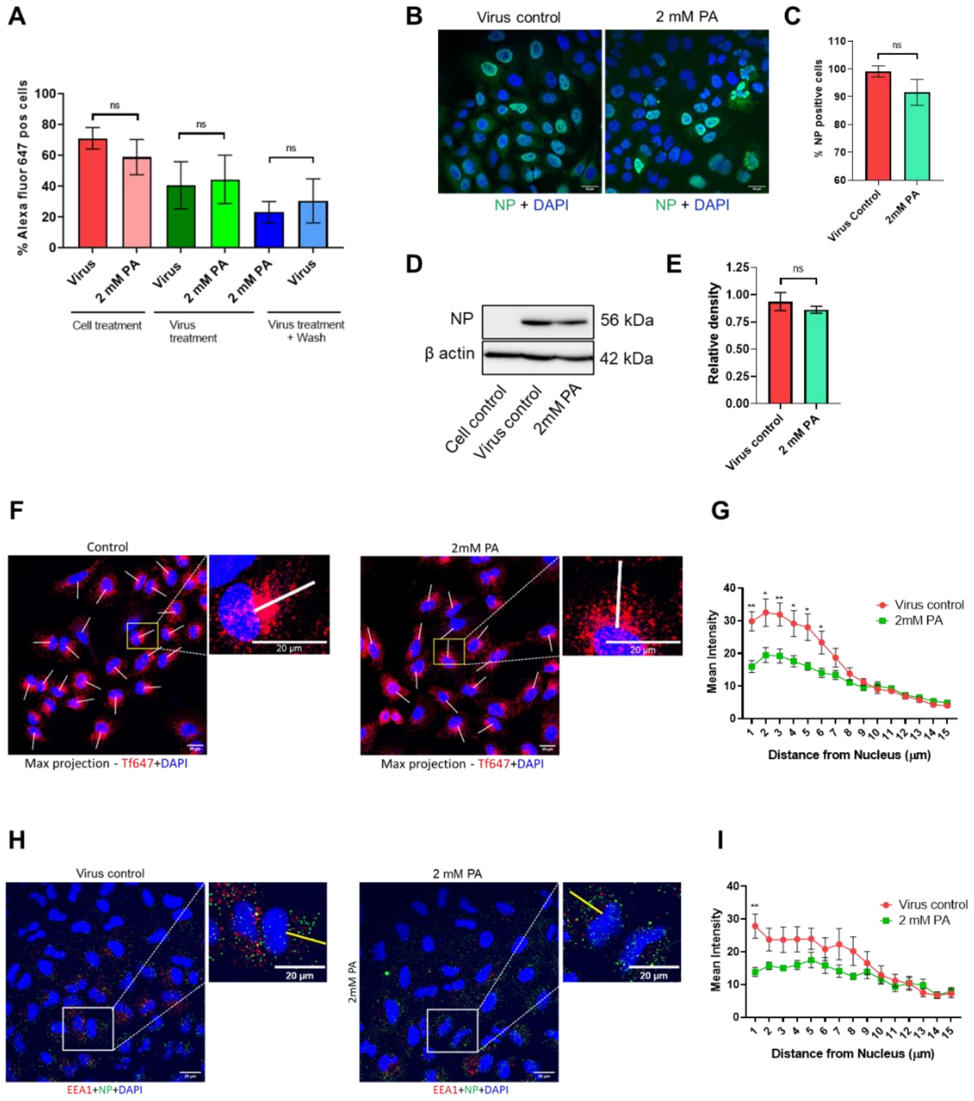

Fig S3. PA treatment does not inhibit virion binding to the host cell but impedes its endocytic movement toward the nucleus. Related to Figure 3. (A) A549 cells suspension or virus inoculum were treated with 2 mM PA under different conditions. Cells were pre-treated for 3hr with 2 mM PA, washed, and infected with 50 MOI PR8 IAV for 60 min on ice in the absence of the drug. Virus inoculum (50 MOI PR8 IAV) was treated with 2 mM PA for 1 hr and used for infection on ice, or the PA-treated inoculum was washed by ultracentrifugation to remove the drug and then used for infection. In all conditions, cells were infected on ice for 1 hr, washed and fixed with 4% PFA and used for HA surface labelling. **(B-E)** A549 cells were infected with 10 MOI PR8 IAV for 1 hr on ice, washed, and warm media containing 2 mM PA was added. After 3 hr, cells were either fixed with 4% PFA for IFA analysis of NP-positive cells, or cell lysates were collected and analyzed by western blot to estimate viral NP expression. (B) Confocal images are showing viral NP-positive cells in green and quantified in (C). (D,E) show corresponding western blot data and quantification of bands, respectively. **(F,G)** A549 cells pre-treated with 2 mM PA were infected with 10 MOI PR8 IAV on ice for 60

min, washed, and incubated at 37°C for another 60 min in the presence of the drug before fixing for IFA. (F) Confocal images show viral NP and cellular EEA-1 in green and red, respectively. (G) Line ROIs were drawn from the nucleus to the cell periphery and fluorescence intensity of Tf-647 was quantified along the length using Leica LasX imaging software. **(H, I)** A549 cells pre-treated for 3 hr with 2 mM PA were pulsed with 25 µg/mL Tf-647 for 30 min, washed, and chased for 15 min in the presence of the drug. (H) Confocal microscopy images showing Tf-647 labeled vesicles in A549 control and 2 mM PA treated cells. (I) Line ROIs were drawn from the nucleus to the cell periphery and the fluorescence intensity of Tf-647 was quantified along the length using Leica LasX imaging software. Scale bar = 20µm. (A) is from 2 independent experiments. All other data are from 3 independent experiments. Datasets include 2-3 technical replicates. *$p < 0.05$, **$p < 0.01$, ns – nonsignificant using a two-tailed unpaired *t*-test or one-way ANOVA with Dunnett's multiple comparison test wherever necessary. Error bars represent mean ± SD.

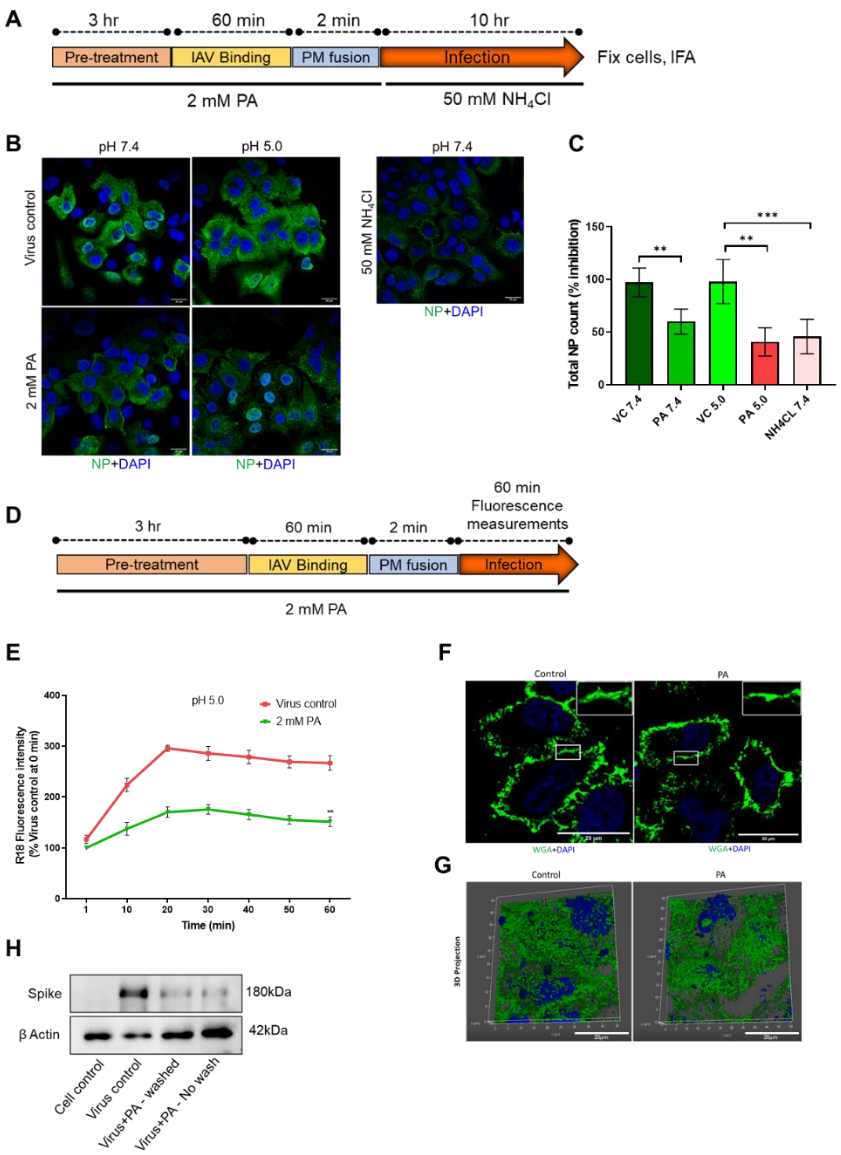

Fig S4. PA inhibits ph5.0-induced fusion of IAV with the plasma membrane, does not affect cell membrane architecture but has an irreversible effect on treated virion infectivity. Related to Figure 3. (A) Schematic showing methodology for endocytosis bypass assay. **(B)** A549 cells pre-treated with 2 mM PA or 50 mM NH_4Cl were infected with 10 MOI PR8 IAV on ice for 60 min. Cells were washed and incubated with either pH 7.4 or pH 5.0 medium for 2 min at 37°C to induce PM fusion. Cells were washed again and incubated for 10 hr with DMEM containing 50 mM NH_4Cl. Negative control of PM fusion used 50 mM NH4Cl at pH 7.4. Cells were then fixed and processed for IFA. Confocal images show NP in green and nuclei in blue. (C) The graph shows the percentage of NP count normalized to virus controls. Quantification was done using ImageJ/Fiji. **(D-E)** R-18 labeled PR8 IAV particles were induced to fuse at the PM in MDCK cells, and the increase in fluorescence intensity upon virus-endosome fusion was measured. (D) Shows a schematic of the

methodology and (E) shows the fluorescence intensity profile up to 60 min. **(F-G)** A549 cells were pre-treated with 2 mM PA for 3hr, fixed with 4% PFA, cell membrane labeled with wheat germ agglutinin (Green), and nuclei with DAPI (Blue). (F) Super-resolution images were acquired using a Leica Stellaris confocal microscope. (G) 3D surface projections created from confocal image z-stacks. Scale bar = 20 μm. **(H)** SARS-CoV-2 virus particles were treated with 2 mM PA for 1 hr at 37°C, washed in PBS for 5 min, ultracentrifuged, and used for infection in Vero E6 cells at 10 MOI. The control virus was not washed and used directly for infection after treatment. Cell lysates were collected at 3 hpi and spike expression was analyzed by western blot. A-E are from 3 independent biological replicates, with datasets including 2-3 technical replicates. F and G are from 1 experiment. H is from 3 independent experiments with technical triplicates. ***$p < 0.001$, **$p < 0.01$ using a two-tailed unpaired *t*-test or one-way ANOVA, wherever necessary. Error bars represent mean ± SD.

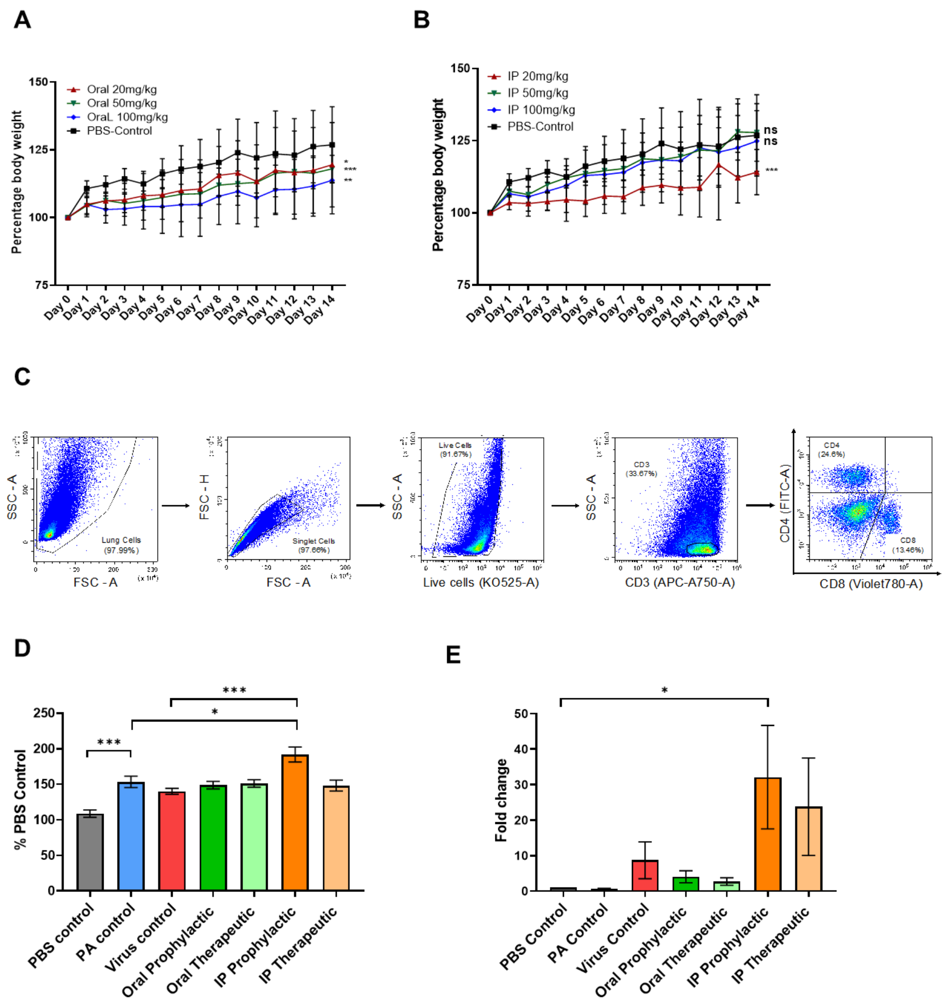

Fig. S5. *In vivo* toxicity profile and immunomodulatory effects of PA in BALB/c mice. Related to Figure 5. Toxicity results as shown by bodyweight changes over 14 days post-treatment with 20, 50, or 100mg/kg PA delivered via **(A)** IP and **(B)** oral routes. Results represent the mean percentage of body weight at Day 0, n= 5 for all groups except n=4 in (A) IP 100mg/kg. **(C-E)** IAV-infected mice, treated with prophylactic and therapeutic dosage regimens of 20 mg/kg PA, were sacrificed 4 dpi, and lungs were analyzed by FACS. (C) Gating strategies to define T cell subsets in lung homogenates. Animal lungs were homogenized, and surface stained with antibodies specific for CD3+, CD4+, and CD8+ markers (D) Quantification of CD4+ T cells from different treatment groups. (E) qRT PCR data showing MIP-1 alpha RNA levels. n=5 in PBS control, PA control, and Oral therapeutic groups and n=4 in all other groups. Data are from 1 experiment. Statistical comparison was done using one-way ANOVA with Bonnferroni's multiple comparisons test. *p< 0.05; **p < 0.01, ***p<0.0001. Error bars indicate mean ± SE.

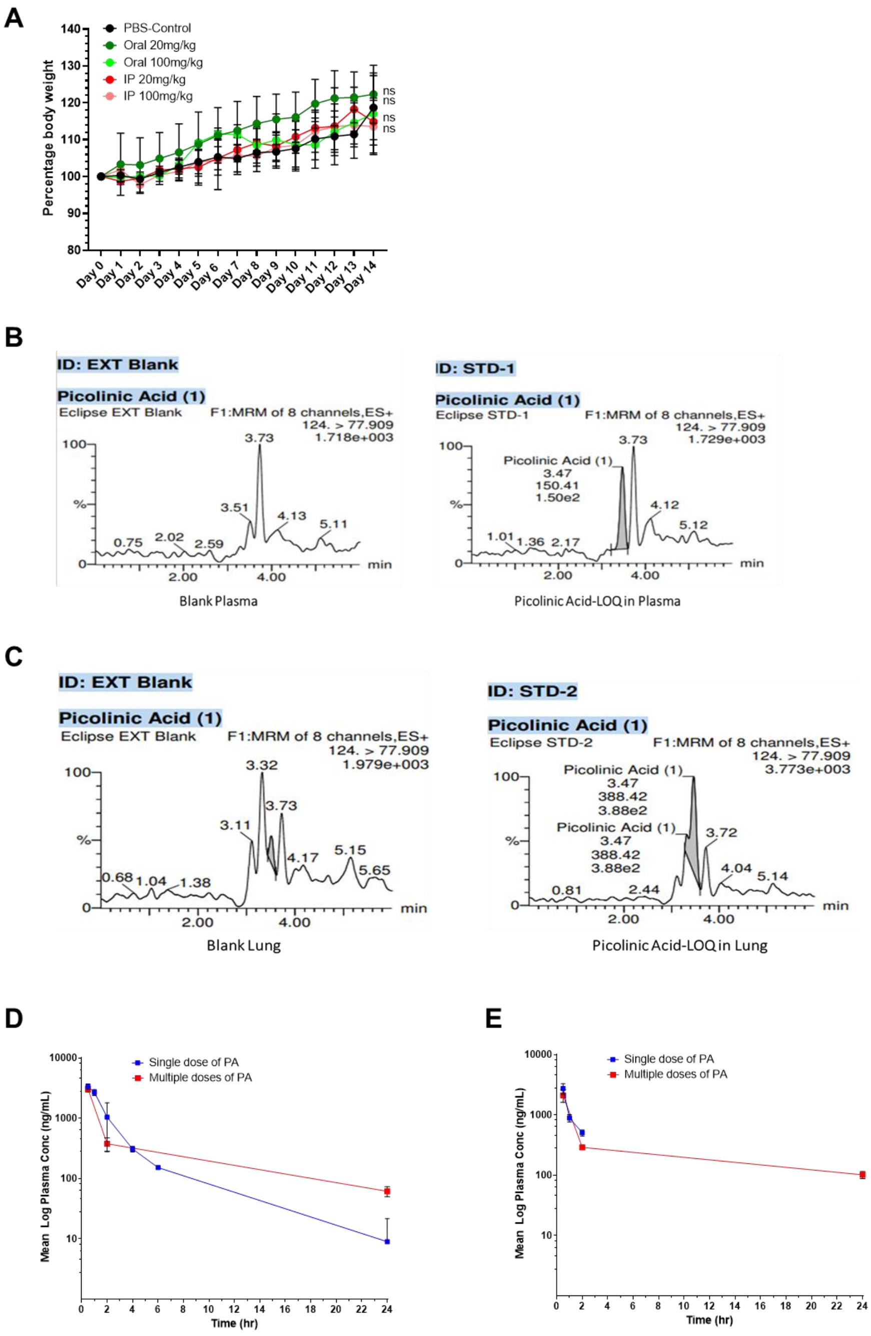

Fig. S6. ***In vivo*** **toxicity profile of PA in Syrian golden Hamsters and pharmacokinetics of PA in animal lungs and blood plasma. Related to Figure 6. (A)** 10-12 weeks old mixed gender Syrian golden hamsters were administered with 20 or 100mg/kg BW via IP or oral routes. Bodyweight was monitored up to day 14 post-treatment. Each group includes 2 males and 2 females. **(B-E)** Hamsters were administered with 20 mg/kg PA orally and levels of the drug in plasma and lungs were estimated by Ultra-Performance Liquid Chromatography. Representational blank plasma and lung sample chromatograms showing the PA identity and the quantification limit are shown in **(B)** and **(C)** respectively. Pharmacokinetics of PA in plasma and lungs following single and

multiple oral doses are shown in **(D)** and **(E),** respectively. (B-E) Each group included n=2 animals. The data shown is from 1 experiment. Statistics for A was done using One-way ANOVA with Dunnett's multiple comparisons. ns: nonsignificant. Error bars represent mean ± SD.
